# A multivariate analysis framework for interpreting long-term zooplankton community dynamics

**DOI:** 10.1093/plankt/fbag082

**Published:** 2026-10-30

**Authors:** Heather L Wander, Mary E Lofton, Jonathan P Doubek, Bryan L Brown, Cayelan C Carey

**Affiliations:** Department of Biological Sciences, University of Quebec at Montréal, 141 Avenue du Président-Kennedy, Montréal, Québec H2X 1Y4, Canada; Department of Biological Sciences, University of Quebec at Montréal and Groupe de Recherche Interuniversitaire en Limnologie, 141 Avenue du Président-Kennedy, Montréal, Québec H2X 1Y4, Canada; Department of Biological Sciences, 926 W Campus Dr., Virginia Tech, Blacksburg, VA 24060, USA; Department of Biological Sciences, 926 W Campus Dr., Virginia Tech, Blacksburg, VA 24060, USA; School of Natural Resources, 650 W. Easterday Avenue, Lake Superior State University, Sault Ste. Marie, MI 49783, USA; Center for Freshwater Research and Education, 100 Salmon Run Way, Lake Superior State University, Sault Ste. Marie, MI 49783, USA; Department of Biological Sciences, 926 W Campus Dr., Virginia Tech, Blacksburg, VA 24060, USA; Department of Biological Sciences, 926 W Campus Dr., Virginia Tech, Blacksburg, VA 24060, USA

**Keywords:** indicator species analysis, non-metric multidimensional scaling, ordination, redundancy analysis, reservoir

## Abstract

Multivariate analyses are widely used to characterize plankton community dynamics in long-term datasets, which often exhibit sparse sampling. These datasets typically require multiple multivariate approaches to describe patterns in community structure, relate those patterns to environmental variation, and identify the taxa contributing to observed differences. However, it can be challenging for plankton researchers to know which multivariate approach to use to best analyze their data. In this paper, our goal was to demonstrate how commonly used multivariate approaches that are typically applied individually can be integrated to provide complementary insights into plankton community variability through time. We applied three approaches—unconstrained ordination, constrained ordination and taxon-specific analyses—to a non-consecutive 7-year freshwater zooplankton dataset to evaluate community variability. Unconstrained ordination revealed variable dynamics within and among years, with the importance of environmental drivers differing within versus among years. Constrained ordination identified several drivers of the zooplankton community, including Secchi depth, total phosphorus, and Schmidt stability. Lastly, taxon-specific analyses identified both the taxa associated with individual years and those driving among-year compositional differences. Our results demonstrate that diverse multivariate methods can provide complementary insights, and sporadic plankton time series can yield robust ecological inferences when analyzed in an integrative framework.

## INTRODUCTION

Zooplankton communities play integral roles in freshwater ecosystems, as they mediate interactions between trophic levels as grazers of phytoplankton and prey for fish (Carpenter *et al.*, 1985; Jeppesen *et al.*, 2011). Numerous studies have demonstrated that freshwater zooplankton communities are variable across temporal scales, from diel to interannual time scales (Rusak *et al.*, 2002; Adrian *et al.*, 2006; Shurin *et al.*, 2010; Li *et al.*, 2019b; Conte *et al.*, 2024; Wander *et al.*, 2024b). This variability has been observed across lakes and reservoirs globally, emphasizing the difficulty in characterizing community dynamics in natural ecosystems (Beaver and Havens, 1996; Bernot *et al.*, 2004; Shurin *et al.*, 2010).

Understanding and predicting zooplankton community responses often requires integrating multiple methodological approaches (Hessen *et al.*, 2006; Legendre and Legendre, 2012; De Senerpont Domis *et al.*, 2013; Downing *et al.*, 2014; Tomljanović *et al.*, 2025). The need for multiple approaches exists because zooplankton dynamics are shaped by nonlinear interactions among environmental drivers, trophic processes, and life-history traits (Lampert, 1989; Sommer *et al.*, 2012; De Senerpont Domis *et al.*, 2013), and each method has its own scope and limitations that govern how well suited it is to a particular question or dataset. For plankton ecologists specifically, the challenge of choosing a suitable analytical approach is often exacerbated because many zooplankton datasets have temporal gaps due to the complex logistics and time-intensive nature of collecting and processing samples. Therefore, several freshwater zooplankton studies have employed a range of different approaches to characterize how communities change over time (Carpenter *et al.*, 2001; Helenius *et al.*, 2015; Mwagona *et al.*, 2018; Kruk *et al.*, 2022), with the suitability of a given approach dependent on both research questions and data availability.

Choosing appropriate analytical approaches for zooplankton time series in freshwater ecosystems is important, as zooplankton variability can be affected by seasonal forcing and hydrological conditions (Geraldes and Boavida, 2005; Cabral *et al.*, 2019; Doubek *et al.*, 2019b). For example, variability in hydrologic conditions due to management practices is one important distinguishing feature of reservoirs versus natural lakes (Hayes *et al.*, 2017), necessitating careful consideration when analyzing reservoir zooplankton time series. Additionally, because many reservoirs are located at the top of their catchment and are very sensitive to meteorological forcing (Thornton *et al.*, 1990; Hayes *et al.*, 2017), intra-annual variability in zooplankton communities may exceed interannual variability, and seasonal succession may deviate from the patterns predicted by models developed for natural lakes (e.g. plankton ecology group (PEG) model; Sommer *et al.*, 1986). While thermal stratification and seasonal nutrient cycling may drive predictable patterns of zooplankton succession in some dimictic temperate reservoirs (Sommer *et al.*, 1986; Wetzel, 2001), reservoir-specific processes such as water level fluctuations driven by climate variability (e.g. changes in precipitation and wind) and management can disrupt these patterns through changes in hydrology, water quality, and trophic interactions (Geraldes and Boavida, 2005; Matveev and Matveeva, 2005). Meteorological conditions, including wind, temperature, and precipitation, can mediate these dynamics indirectly by influencing thermal stratification, nutrient loading, and primary production, which in turn can further increase variability in zooplankton community structure (Gyllström *et al.*, 2005; Rinke *et al.*, 2009; Gürbüzer *et al.*, 2026).

Given that zooplankton communities respond to environmental conditions differently across spatial and temporal scales, multivariate statistical approaches have much potential for analyzing zooplankton community dynamics to yield ecological insights on community variability and taxon-specific responses (Pinel-Alloul *et al.*, 1995; Helenius *et al.*, 2015; Mwagona *et al.*, 2018). Multivariate approaches treat each sampling event as an independent multivariate observation and do not require evenly spaced or contiguous time series, allowing for the characterization of zooplankton dynamics when observations are sparse or irregularly sampled (Obertegger *et al.*, 2010; Aguilera *et al.*, 2016; Gong *et al.*, 2021; Tomljanović *et al.*, 2025). Integrative studies using several multivariate analyses can provide complementary perspectives on zooplankton community dynamics by contrasting unconstrained patterns of community structure with environmentally constrained and taxon-specific responses (Yang *et al.*, 2018; Napiórkowski *et al.*, 2019; Li *et al.*, 2019a; Qiu *et al.*, 2022). For example, Hu *et al.* (2019) used non-metric multidimensional scaling (NMDS) to visualize seasonal patterns in zooplankton community structure, indicator species analysis (ISA) to identify which zooplankton groups were driving the observed patterns in community structure, and redundancy analysis (RDA) to identify significant relationships between the environmental drivers and dominant species within the zooplankton community. These multiple analyses provided complementary insights on seasonal shifts in zooplankton community structure by linking community variability, taxonomic dominance, and environmental drivers, highlighting the utility of using integrated multivariate analyses. However, to our knowledge, studies have yet to consider how the choice of differing multivariate approaches can provide complementary or contrasting ecological information on zooplankton community structure, providing an opportunity to examine the inferences that can be made from complementary analyses applied to a representative zooplankton dataset. For example, while Hu *et al.* (2019) used ISA to identify indicator species, there are other methods that they may have used for this purpose (e.g. similarity percentages and threshold species taxa analysis), which may have yielded different results. Comparison of multiple multivariate methods that are commonly used with the same aim in mind (e.g. identification of indicator species) can assist zooplankton researchers in selecting which method may be appropriate for their research study.

Here, our goal was to assess how multivariate methodological choice influences ecological interpretation of zooplankton communities. Although multispecies surveys have examined zooplankton responses to environmental and spatial drivers (e.g. Beisner *et al.*, 2006), direct comparisons of multiple multivariate community analysis methods remain rare for zooplankton (e.g. RDA vs. distance-based RDA (dbRDA) rather than multiple independent analyses). Methodological assessments of different multivariate approaches that focus on high-resolution or continuous time series (Angeler *et al.*, 2009) or microbial community datasets (Kuczynski *et al.*, 2010) have been explored. However, these assessments typically rely on complete or finely resolved time-series data and therefore do not address the challenges posed by the incomplete or irregular sampling typical of zooplankton datasets. As a result, there remains little guidance on how different multivariate approaches influence ecological inference when applied to the discontinuous, multi-year datasets that are common in plankton research.

In this study, we integrated unconstrained NMDS, constrained RDA, and taxon-specific analyses to evaluate temporal trends in the zooplankton community over seven non-consecutive years in a eutrophic reservoir that experiences substantial seasonal, hydrological, and meteorological variability over time. Because variability in zooplankton communities can occur over multiple timescales (e.g. Simões *et al.*, 2012; Brown *et al.*, 2016), there is a need for studies that consider both intra- and interannual changes in freshwater zooplankton community dynamics. We compared results from two unconstrained ordination methods, two constrained ordination methods, and two taxon-specific multivariate methods. Together, these methods combine community-level patterns and taxon-specific responses, improving interpretation of how zooplankton communities respond to environmental variability. We used these analyses to answer two research questions: (1) How do zooplankton community structure and key environmental drivers vary across months and years? and (2) What insights on zooplankton community variability can we gain from using multiple multivariate approaches within a single ecosystem? We expected that hydrological changes in water level would favor greater zooplankton community variability within years rather than between years, and that seasonal changes in temperature and nutrients would be important environmental drivers of zooplankton community structure across most years. Ultimately, our goals were to both advance understanding of zooplankton community variability in a eutrophic reservoir over multiple years and to demonstrate the integration of complementary multivariate approaches as a practical guide for plankton ecologists working with similar datasets.

## METHODS

### Study site

Beaverdam Reservoir (BVR) is a eutrophic reservoir located in a completely forested catchment in Vinton, VA, USA. BVR is owned and managed by the Western Virginia Water Authority, has a maximum depth of 13.4 m, and has a surface area of 0.38 km^2^ (Hamre *et al.*, 2018; Doubek *et al.*, 2019a). During the stratified period (~April–November), BVR experiences bottom-water anoxia, in which dissolved oxygen (DO) concentrations are < 1 mg/L, and a deep-water chlorophyll *a* maximum develops in most years (Hamre *et al.*, 2018). Throughout the study, BVR water level fluctuated between 7.9 and 13.3 m (Carey *et al.*, 2024a), with a managed drawdown during the summer stratified period in 2022, during which water level ranged between 7.9 and 8.1 m (Lewis *et al.*, 2024). Outside of water level fluctuations, the food web was assumed to be relatively stable with no changes in land use or other food web processes.

### Field sampling

We collected weekly to monthly zooplankton samples in BVR from May to October 2014–2016, 2019–2021 and 2023, with occasional sampling in March, April, November and December some years (Fig. S1). Therefore, our analysis of zooplankton community data is limited to the thermally stratified period, as we had limited zooplankton samples collected during the mixed period. We collected a total of *n* = 85 zooplankton samples during the study period. Full water column tows (8–10 m, depending on water level) were collected using an 80-μm Wisconsin-style zooplankton net (diameter = 0.3 m, length = 0.95 m; Wildlife Supply Company, Yulee, FL, USA) at the deepest site in BVR, where long-term sampling occurred (37.313° N, 79.816° W). Net tow samples were collected from ~ 1 m above the sediments to the surface of the reservoir, preserved in ~ 70% ethanol by volume in the field, and stored in the laboratory until processing occurred. While nets with smaller mesh sizes are typically used when collecting rotifers, BVR is a eutrophic reservoir and therefore has lower rates of net efficiency due to clogging with smaller mesh sizes (Lathrop, 1998), necessitating a net with a larger mesh size (Wander *et al.*, 2024b). We realize some smaller rotifer taxa were likely not captured by our zooplankton nets, but this effect was consistent across all samples collected. Therefore, we limit our interpretation of rotifer community dynamics to large rotifers only in this manuscript. To correct for net efficiency in our samples, we collected Schindler trap samples (30 L, 61-μm mesh) at 1-m intervals throughout the entire water column on 8 days alongside the zooplankton net tows during the study period (2016, 2019, 2020, and 2021), following St. Amand *et al.* (1998). Schindler traps capture discrete water volumes without the filtration inefficiencies associated with towed nets, allowing us to calculate a correction factor for the zooplankton net tow samples (see Wander *et al.*, 2024b for more details and R code used for the net efficiency calculations).

In addition to zooplankton samples, we also collected physical, chemical, and biological data at BVR’s deepest site. We collected water temperature and DO concentration profiles using a Sea-Bird Conductivity, Temperature, and Depth (CTD) profiler (Sea-Bird Scientific, Bellevue, WA, USA; Carey *et al.*, 2024d). During the few days that the CTD profiler was not available, we measured temperature and DO concentration using a YSI handheld probe (ProPlus with Quattro cable or ProODO optical dissolved oxygen meter, YSI Inc., Yellow Springs, OH, USA; Carey *et al.*, 2024c). Comparisons between paired CTD and YSI depth profiles indicate that both methods yield similar temperature and DO data (Carey *et al.*, 2022a). Secchi depth measurements were obtained on each sampling day (Carey *et al.*, 2024c). In addition, we collected and analyzed dissolved and total nutrient (nitrogen, phosphorus) samples from the surface and bottom-waters (Carey *et al.*, 2024e) following methods described by Carey *et al.* (2022a). Phytoplankton community data for four spectral groups were collected using a FluoroProbe (bbe Moldaenke, Schwentinental, Germany; Carey *et al.*, 2024b). Nutrient, water temperature, and DO data were binned into epilimnetic and hypolimnetic means, which were calculated by averaging all values above (epilimnetic) versus below (hypolimnetic) the daily thermocline. Physical reservoir metrics were calculated from field data, including thermocline depth, oxycline depth, and Schmidt stability using the *rLakeAnalyzer* package (Winslow *et al.*, 2019). Residence time was calculated by dividing daily reservoir volume (Carey et al., 2022b) by an inflow rate estimated from meteorology and soil characteristics (Woelmer *et al.*, 2022; Wander *et al.*, 2025).

We used hourly meteorological data from the North American Land Data Assimilation System (NLDAS-2), which was precipitation corrected (Xia *et al.*, 2012). These data included air temperature (°C), shortwave radiation (W m^−2^), longwave radiation (W m^−2^), wind speed (m s^−1^), precipitation (m d^−1^), and relative humidity (%). Hourly data were aggregated to daily means for each of the sampling days when zooplankton were collected.

### Zooplankton processing

All zooplankton samples were processed using a Meiji RZ dissecting microscope (Meiji Techno, San Jose, CA, USA) following standard methods (Downing and Rigler, 1984; McCauley, 1984). We counted at least 3 aliquots or 100 individual zooplankton per sample. While 100 individuals per sample may underrepresent rare taxa, we prioritized counting consistency across sampling years to maximize temporal coverage (Carey *et al.*, 2026). Cladocera and Rotifera were identified to genus and Copepoda were identified to order (Carey *et al.*, 2026). We recognize that the broad identification of copepods (as either Calanoida, Cyclopoida, or nauplii) is a limitation of our study; however, this broad identification is common across zooplankton datasets (e.g. Kumari *et al.*, 2023; Glandon *et al.*, 2024; Meredith *et al.*, 2025). Although a reduced taxonomic resolution existed for Copepoda relative to Cladocera and Rotifera, this approach provided a consistent basis for temporal comparisons across the full dataset. Code for calculating zooplankton density is published in the Zenodo repository (Wander *et al.*, 2024a).

### Applying multivariate analyses to zooplankton community data

To characterize patterns in zooplankton community composition and identify potential environmental drivers of zooplankton in BVR, we applied a combination of unconstrained, constrained, and species-level multivariate approaches. For NMDS, we selected the common zooplankton taxa that were consistently present across study years, resulting in 10 distinct taxa. These 10 taxa comprised 96% of total zooplankton abundance by density over our study period. We used the 10 dominant taxa for NMDS, as these analyses were intended to visualize high-level changes in zooplankton community composition over time. For all other multivariate analyses, we used all 23 unique taxa identified, as constrained ordinations and taxon-specific analyses are well suited to community datasets containing rare or infrequently occurring taxa (McCune and Grace, 2002; Legendre and Legendre, 2012). All multivariate analyses were performed in R (v.4.5.2) using the *vegan* and *indicspecies* R packages (De Cáceres and Legendre, 2009; Oksanen *et al.*, 2022).

### Unconstrained ordinations

We used NMDS based on Bray–Curtis dissimilarities to visualize overall patterns in community structure. NMDS is a rank-based ordination method with flexibility in the choice of distance metrics used to represent patterns in multivariate ecological community data (Kruskal, 1964; Clarke, 1999). We used a scree plot to determine the optimal number of dimensions (*k*), balancing model complexity against goodness of fit (McCune and Grace, 2002). All 85 zooplankton samples were used for our first-stage NMDS application. We calculated Euclidean distances between each pair of year centroids across all NMDS axes to determine years with the most similar zooplankton community structure, where similarity was identified as years with short Euclidean distances. Because April and November were not sampled in every year, year centroids at this stage reflect only the months available for a given year, and these results are interpreted descriptively rather than using a formal test of difference among years. To evaluate temporal differences in zooplankton community composition, we first tested for homogeneity of multivariate dispersion using *betadisper*() from the *vegan* package (Oksanen *et al.*, 2022). Because permutational multivariate analysis of variance (PERMANOVA) can be sensitive to differences in within-group dispersion, this analysis was used to verify that any detected differences in community composition reflected shifts in group centroids rather than unequal dispersion. Both *betadisper* and the subsequent PERMANOVA tests were conducted using a balanced subset of samples restricted to May–October, which were the months sampled consistently across all seven non-consecutive study years (2014, 2015, 2016, 2019, 2020, 2021, and 2023). Restricting the analysis to these consistently sampled months minimized unequal sample sizes and prevented seasonal differences in sampling coverage from confounding comparisons among years.

To examine higher-level temporal patterns in community structure, we used second-stage NMDS to visualize patterns in community composition based on a group-level dissimilarity matrix derived from the first-stage community distances (Clarke *et al.*, 2006). Second-stage NMDS uses correlations between first-stage dissimilarity matrices to condense community-level variation into a single ordination, allowing among-group patterns to be visualized more clearly. Specifically, we subsetted the dataset to only include samples that were collected between May and October, as second-stage NMDS requires an even number of samples across years (Clarke *et al.*, 2006). Therefore, 73 samples that were collected in May–October were aggregated into monthly averages for our second-stage NMDS application (analyzing 6 months of aggregated means × 7 years of data). Significant environmental variables were fitted onto the NMDS ordination using the *envfit*() function (*vegan* package), where only variables with significant correlations with the ordination axes (*P* < 0.05) were visualized. Zooplankton density data were Hellinger transformed for both first- and second-stage NMDS applications. Environmental data (see below) were imputed using linear interpolation and monthly means for both NMDS applications and aggregated to the monthly scale for second-stage NMDS.

### Constrained ordinations

To evaluate relationships between environmental variables and zooplankton community composition, we applied both RDA and dbRDA. RDA is a constrained ordination technique that directly relates multivariate community composition to measured environmental variables by combining multiple regression and principal component analysis (ter Braak, 1990; Legendre and Legendre, 2012). Distance-based redundancy analysis (e.g. dbRDA) extends this framework by allowing community dissimilarities to be analyzed using alternative distance metrics when ecological distances are not well represented by Euclidean space (Legendre and Anderson, 1999). We used detrended correspondence analysis (DCA) to evaluate gradient lengths, which were short (<3 SD units), indicating linear species responses and justifying the use of RDA in our dataset (Legendre and Legendre, 2012). If gradients were long or unimodal relationships were expected, canonical correspondence analysis (CCA) would be more appropriate.

We first evaluated gradient lengths using DCA and found that gradient lengths were short, indicating that taxon responses to the environmental variables were adequately approximated by linear models; we therefore used RDA rather than CCA, which is designed for unimodal responses along longer gradients. We performed an RDA on Hellinger-transformed density data to quantify the proportion of variance in community composition explained by environmental gradients. Then, we applied a dbRDA (Legendre and Anderson, 1999) using Bray–Curtis dissimilarities to account for nonlinear species–environment relationships and compositional turnover. We used Procrustes analyses (*procrustes*() and *protest*() with 999 permutations in the *vegan* package) to determine if dbRDA and RDA ordinations were more similar than expected by chance (Oksanen *et al.*, 2022). This approach allowed us to evaluate the robustness of our findings, as agreement between methods would strengthen confidence in the environmental variables most strongly associated with zooplankton community structure. Both RDA and dbRDA were conducted on the full dataset of 85 zooplankton samples collected between April and November over 7 non-consecutive years using imputed environmental driver data (see below). Model significance was assessed using permutation-based ANOVA (999 permutations) to test (1) the significance of individual canonical axes and (2) the unique (marginal, Type III) contribution of each environmental variable after controlling for all other variables in the model. We removed colinear environmental variables with variance inflation factors > 5 and only visualized significant environmental variables in ordination plots. To partition explained variation among environmental predictors, we grouped variables into physical, chemical, and meteorological groups and performed variation partitioning using the *varpart*() function in the *vegan* package. Adjusted *R*^2^ values were used to quantify the unique and shared contributions of each group to zooplankton community variance.

### Taxon-specific analyses

Lastly, we used two complementary taxon-specific analyses. First, we used ISA to identify taxa that were significantly associated with particular years in our dataset (Dufrêne and Legendre, 1997). ISA assumes that groups are ecologically meaningful and sufficiently sampled, and that species occurrences are reasonably representative of the communities studied (Dufrêne and Legendre, 1997; De Cáceres *et al.*, 2010). For each taxon, an indicator value was calculated based on its specificity (uniqueness to a group) and fidelity (frequency within that group), with values approaching zero indicating no group association (Dufrêne and Legendre, 1997). Unlike approaches that identify taxa based on maximum densities alone, ISA accounts for both the uniqueness of a taxon to a particular year and its consistency of occurrence within that year, making it more robust for identifying reliable biological indicators of interannual community shifts. Statistical significance was assessed using permutation tests that compared observed indicator values against a null distribution generated by random reassignment of taxa to groups (Dufrêne and Legendre, 1997; De Cáceres and Legendre, 2009). Second, we used a similarity percentage (SIMPER) analysis to identify which taxa contributed most to between-year compositional differences. SIMPER provided a descriptive decomposition of average dissimilarity between groups (e.g. years) by identifying taxa that contributed most to compositional differences (Clarke, 1993). Mean contribution values were averaged across all pairwise year comparisons for each taxon and taxa were retained up to a cumulative 70% of total mean contribution. Together, ISA and SIMPER provided complementary perspectives: ISA identified taxa that were consistently associated with particular years, while SIMPER highlighted taxa that most strongly contributed to differences in community composition between those years. We used Hellinger-transformed density data for ISA, as recommended for community abundance data to reduce the influence of dominant taxa and rare-species double-zero effects (De Cáceres and Legendre, 2009), and raw density data for SIMPER following the standard implementation using Bray–Curtis dissimilarity (Clarke, 1993).

### Environmental driver analysis

As described above, we used environmental drivers in our NMDS and RDA applications to identify relationships between physical and chemical variables and zooplankton community data over the study period (see environmental variable summary Table S1). Missing values in environmental drivers were addressed in two ways. First, values were linearly interpolated within each calendar year so long as the gaps between observations were ≤15 days. Second, any remaining missing values were replaced by the monthly mean across all years for that variable. For second-stage NMDS, daily environmental variables (air temperature, relative humidity, precipitation, wind speed, and shortwave and longwave radiation) were averaged to the monthly scale to match the aggregation in the zooplankton dataset.

### Data and code availability

All statistical code is available in the Zenodo repository (Wander *et al.*, 2026), and all zooplankton data are available in the Environmental Data Initiative repository (Carey *et al.*, 2026).

## RESULTS

We identified 23 unique zooplankton taxa in BVR over the study period (Table S2). Of the 23 taxa, 10 taxa were consistently observed each year (in order of most to least dominant, by density): copepod nauplii, *Kellicottia*, *Keratella*, *Conochilus*, Cyclopoida, *Polyarthra*, *Bosmina*, *Daphnia*, *Ceriodaphnia*, and *Trichocerca*. The remaining zooplankton taxa were observed on <35% of sampling days (Table S2); therefore, only the density patterns of the more frequently observed taxa are described here. *Kellicottia* tended to peak in early June, though maximum densities were variable across years (Fig. 1, Fig. S2). *Conochilus* and *Daphnia* density patterns were similarly variable across years, with mean peaks at the end of June. *Keratella* density was greatest between June and August, except for 2023, where density was greatest in April (Fig. 1, Fig. S2). Copepod nauplii and *Ceriodaphnia* density tended to peak in July, ranging between June and September most years, whereas Cyclopoida density tended to peak in August, but ranged from June to November across years (Fig. 1, Fig. S2). *Trichocerca* density peaked between July and October all years except for 2023, where density peaked in April. *Bosmina* density peaks occurred latest in the year, ranging from August to December all years except for 2014, when the maximum density peak occurred at the end of April (Fig. 1, Fig. S2). *Polyarthra* density peaks were highly variable across years, ranging from May to December, with the average density peak in early August over the seven non-consecutive years.

**Fig. 1:**
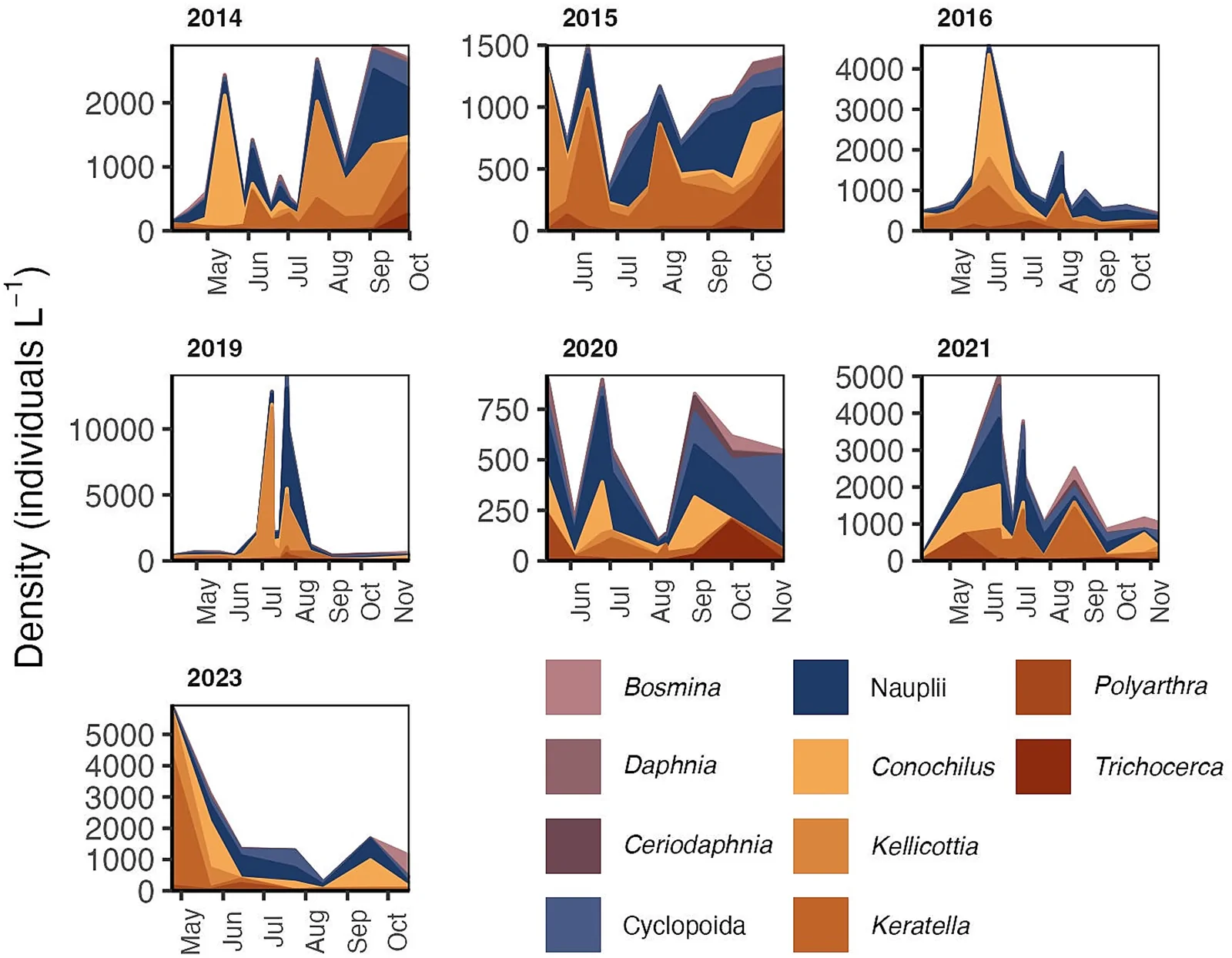
Zooplankton density for the 10 common taxa observed in Beaverdam Reservoir from 2014 to 2023. Note different *x*-axis ranges, which correspond to when the first and last zooplankton samples were taken each year. Panels depict each of the seven non-consecutive years included in this study. Cladocera are shown in mauve, Cyclopoida in blue and Rotifera in orange/brown. Cyclopoida represent order, nauplii are larval copepods and all the other zooplankton taxa are genera. Note varying *y*-axes among years.

### Unconstrained ordination identified significant variation in zooplankton communities across months and years

First-stage NMDS ordinations (*k* = 3, stress = 0.14) highlighted patterns in zooplankton community structure across temporal scales. We found that zooplankton community structure differed across months and years, as indicated by the separation of month and year centroids in the ordination (Fig. 2) and PERMANOVA results (month *R*^2^ = 0.19, *F*_5,67_ = 3.07, *P* = 0.001; year *R*^2^ = 0.20, *F*_6,66_ = 2.77, *P* = 0.001). However, month and year polygons largely overlapped across all three axes, suggesting some similarity in zooplankton community dynamics across these scales (i.e. zooplankton community structure only varied across some pairwise month/year combinations; Fig. 2, Fig. S3). Homogeneity of multivariate dispersion was confirmed for both months (*P* = 0.16) and years (*P* = 0.79), indicating that PERMANOVA results reflect differences in community composition rather than dispersion. *Envfit* results indicated that green algae, blue-green algae, brown algae, and mixed algae pigments were significantly related to NMDS ordination axes (Figs S4–S5; Table S3). Additionally, Secchi depth, epilimnetic dissolved oxygen (DO), hypolimnetic water temperature, hypolimnetic total nitrogen (TN), hypolimnetic total phosphorus (TP), Schmidt stability, and epilimnetic water temperature were also significantly related to NMDS ordination axes (Table S3).

**Fig. 2:**
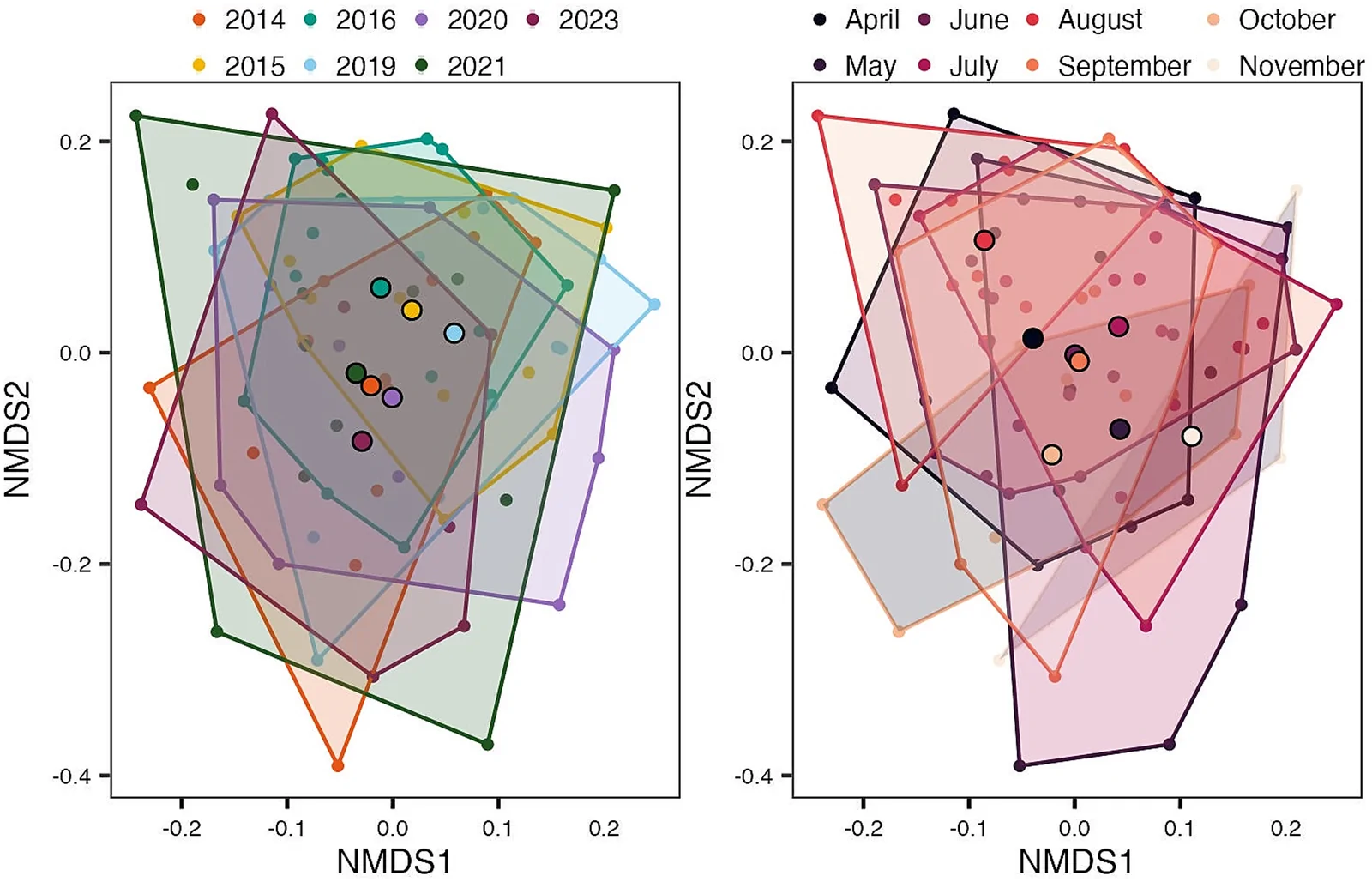
First-stage non-metric multidimensional scaling (NMDS) ordination for axes 2 versus 1 using zooplankton density data from the 10 common taxa observed Beaverdam Reservoir over the seven non-consecutive year study period (*k* = 3; stress = 0.14). Both panels depict the same ordination with different polygon groupings, where points summarize temporal trends in summer community structure data from distinct sampling days. Large colored points represent the centroid of each polygon. Polygons in the left panel correspond to years (*n* = 7) and polygons in the right panel correspond to months (*n* = 8). This ordination summarizes zooplankton data collected from *n* = 85 distinct sampling days. Axes 3 versus 1 are shown in Fig. S3.

Second-stage NMDS (*k* = 3, stress = 0.10) highlighted similarities in intra-annual zooplankton community composition in ordination space. We found that 2021 and 2023 had the most similar May–October zooplankton community structure, as indicated by the smallest Euclidean distance across all three axes (Fig. 3; Table I). The second most similar year pairs were observed for 2015 and 2016, as well as 2020 and 2021, which both had Euclidean distance values of 0.48. Conversely, 2015 and 2020 had the most d istinct May–October zooplankton communities (Fig. 3; Table I). Correlations between environmental variables and second-stage NMDS axes, as determined using *envfit*, indicated that water level and Schmidt stability were associated with second-stage NMDS axes (Fig. S6; Table S4). Specifically, water level and Schmidt stability were positively associated with 2014 and 2015 zooplankton community dynamics (Fig. S6).

**Table I:** Euclidean three-dimensional distances calculated using all three second-stage NMDS dimensions for all seven years of zooplankton density data collected from Beaverdam Reservoir (n = 73)

	2014	2015	2016	2019	2020	2021	2023
2014	0	0.66	0.55	0.57	0.49	0.70	0.88
2015		0	0.48	0.50	0.92	0.84	0.67
2016			0	0.76	0.70	0.85	0.79
2019				0	0.74	0.50	0.55
2020					0	0.48	0.77
2021						0	0.42
2023							0

Smaller values indicate greater similarity between years

**Fig. 3:**
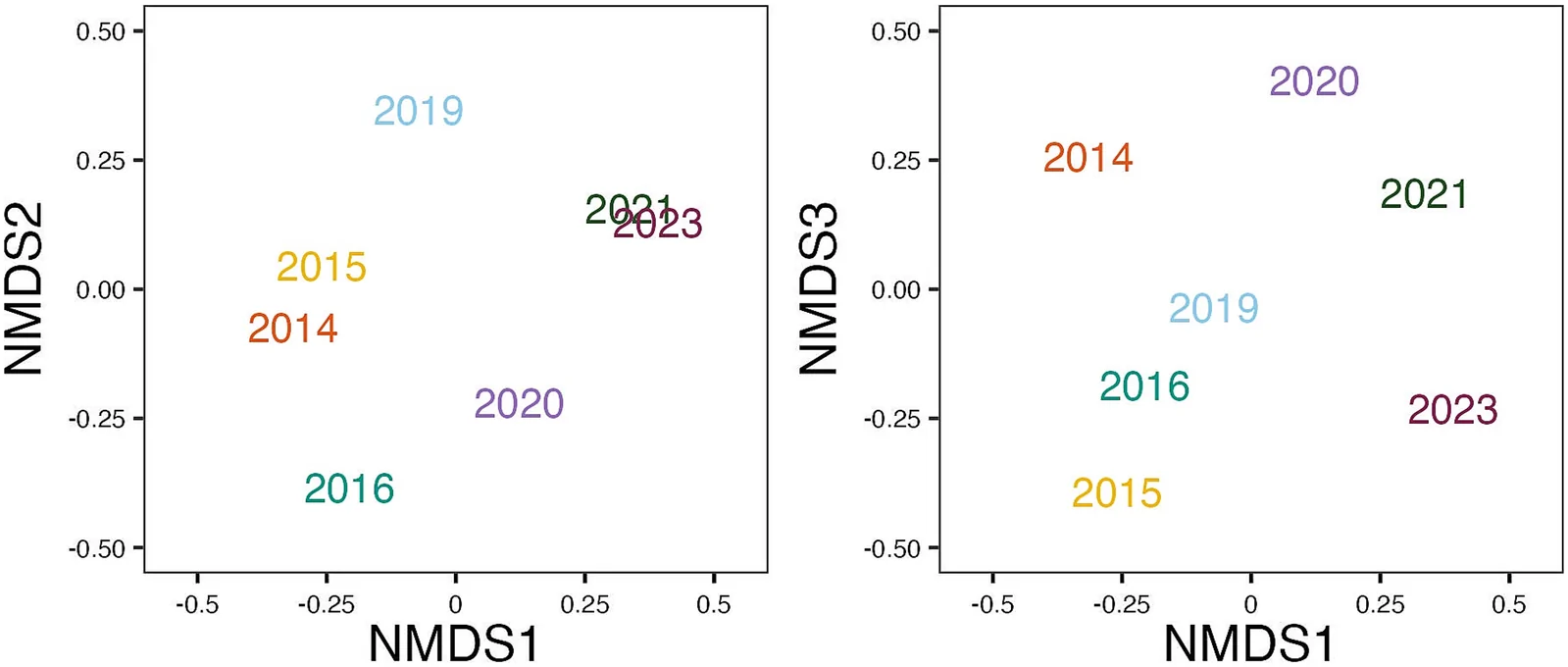
Second-stage non-metric multidimensional scaling (NMDS; *k* = 3, stress = 0.10) calculated using pairwise correlations among all distance matrices for each year of zooplankton density data. Grouping of years indicate similarities in monthly patterns of zooplankton community structure in Beaverdam Reservoir. Left panel corresponds to axis 2 versus axis 1; right panel corresponds to axis 3 versus axis 1. Note that samples were aggregated to the monthly scale for May–October each year to meet the equal sample size requirement for this analysis.

### Constrained ordination highlighted association between thermal stratification and cladoceran and rotifer taxa

Environmental conditions accounted for a meaningful proportion of variability in zooplankton community structure in both RDA and dbRDA analyses (23.2% and 20.7% of total variability explained, respectively), and the two ordinations were highly congruent (Procrustes *r* = 0.97, RMSE = 0.11, *P* = 0.001). Axes 1–2 (RDA) and axes 1–3 (dbRDA) each explained a significant fraction of variance (all *P* < 0.05; Tables S5–S6). Permutation tests of marginal means for both RDA and dbRDA analyses identified broadly consistent environmental drivers of zooplankton community structure (Tables II–III), including Schmidt stability, Secchi depth, water level, hypolimnetic water temperature, thermocline depth, epilimnetic TN, and brown-algae pigments. Variation partitioning indicated that physical and chemical variables each explained 8.4% and 6.2% of zooplankton community variance, respectively, with 5.6% shared between both chemical and physical groups (Table S7). Meteorological variables explained a negligible percentage of the total variation (<0.01).

**Table II:** Permutation test of marginal effects for environmental variables included in the redundancy analysis (RDA)

Environmental variable	*F*-value	*P*-value	Variance explained (%)
Water level (m)	2.88	**0.005**	6.5
Schmidt stability (J/m^2^)	2.90	**0.007**	6.5
Epilimnetic total nitrogen (μg/L)	2.61	**0.011**	5.9
Brown-algae pigments (μg/L)	2.62	**0.016**	5.9
Hypolimnetic water temperature (°C)	2.47	**0.021**	5.6
Secchi depth (m)	2.49	**0.016**	5.6
Thermocline depth (m)	2.27	**0.018**	5.1
Relative humidity (%)	1.87	0.066	4.2
Epilimnetic total phosphorus (μg/L)	1.62	0.115	3.7
Epilimnetic dissolved oxygen (mg/L)	1.65	0.100	3.7
Blue-green (cyanobacteria) pigments (μg/L)	1.31	0.222	3.0
Shortwave radiation (W/m^2^)	1.35	0.211	3.0
Hypolimnetic total phosphorus (μg/L)	1.12	0.331	2.5
Hypolimnetic total nitrogen (μg/L)	0.81	0.585	1.8
Green-algae pigments (μg/L)	0.78	0.623	1.8
Oxycline depth (m)	0.76	0.643	1.7
Mixed algae pigments (μg/L)	0.74	0.656	1.7
Rain (m/day)	0.75	0.654	1.7
Wind speed (m/s)	0.23	0.998	0.5

*F*-values and *P*-values indicate the significance of each variable in explaining zooplankton community composition. Variance explained (%) refers to the proportion of the total constrained variation accounted for by each term. Significant *P*-values are shown in bold. Environmental variables are ordered from greatest to least contribution of explained variance

**Table III:** Permutation test of marginal effects for environmental variables included in the distance-based redundancy analysis (dbRDA)

Environmental variable	*F*-value	*P*-value	Variance (%)
Water level (m)	2.79	**0.004**	6.8
Epilimnetic total nitrogen (μg/L)	2.58	**0.005**	6.3
Brown-algae pigments (μg/L)	2.42	**0.010**	5.9
Hypolimnetic water temperature (°C)	2.20	**0.020**	5.4
Schmidt stability (J/m^2^)	2.21	**0.021**	5.4
Secchi depth (m)	1.88	**0.041**	4.6
Thermocline depth (m)	1.82	**0.039**	4.5
Epilimnetic dissolved oxygen (mg/L)	1.74	0.051	4.3
Blue-green (cyanobacteria) pigments (μg/L)	1.68	0.064	4.1
Relative humidity (%)	1.53	0.117	3.7
Epilimnetic total phosphorus (μg/L)	1.48	0.121	3.6
Hypolimnetic total phosphorus (μg/L)	1.14	0.294	2.8
Shortwave radiation (W/m^2^)	1.15	0.303	2.8
Mixed algae pigments (μg/L)	0.89	0.528	2.2
Hypolimnetic total nitrogen (μg/L)	0.85	0.578	2.1
Rain (m/day)	0.77	0.665	1.9
Green-algae pigments (μg/L)	0.71	0.768	1.7
Oxycline depth (m)	0.66	0.829	1.6
Wind speed (m/s)	0.36	0.994	0.9

*F*-values and *P*-values indicate the significance of each variable in explaining zooplankton community composition. Variance (%) refers to the proportion of the total constrained variation accounted for by each variable. Significant *P*-values are shown in bold. Environmental variables are ordered from greatest to least contribution of explained variance

RDA and dbRDA results revealed five groups of zooplankton taxa with distinct associations with environmental variables (Fig. 4). Thermal stratification, represented by Schmidt stability, was a strong structuring force: *Keratella* was positively associated with stronger stratification strength, while *Bosmina*, *Chydorus, Polyarthra*, *Ascomorpha*, and *Conochilus* were negatively associated. *Kellicottia* was negatively associated with brown-algae pigments, whereas Cyclopoida, nauplii, and *Ceriodaphnia* were positively associated with hypolimnetic water temperature.

**Fig. 4:**
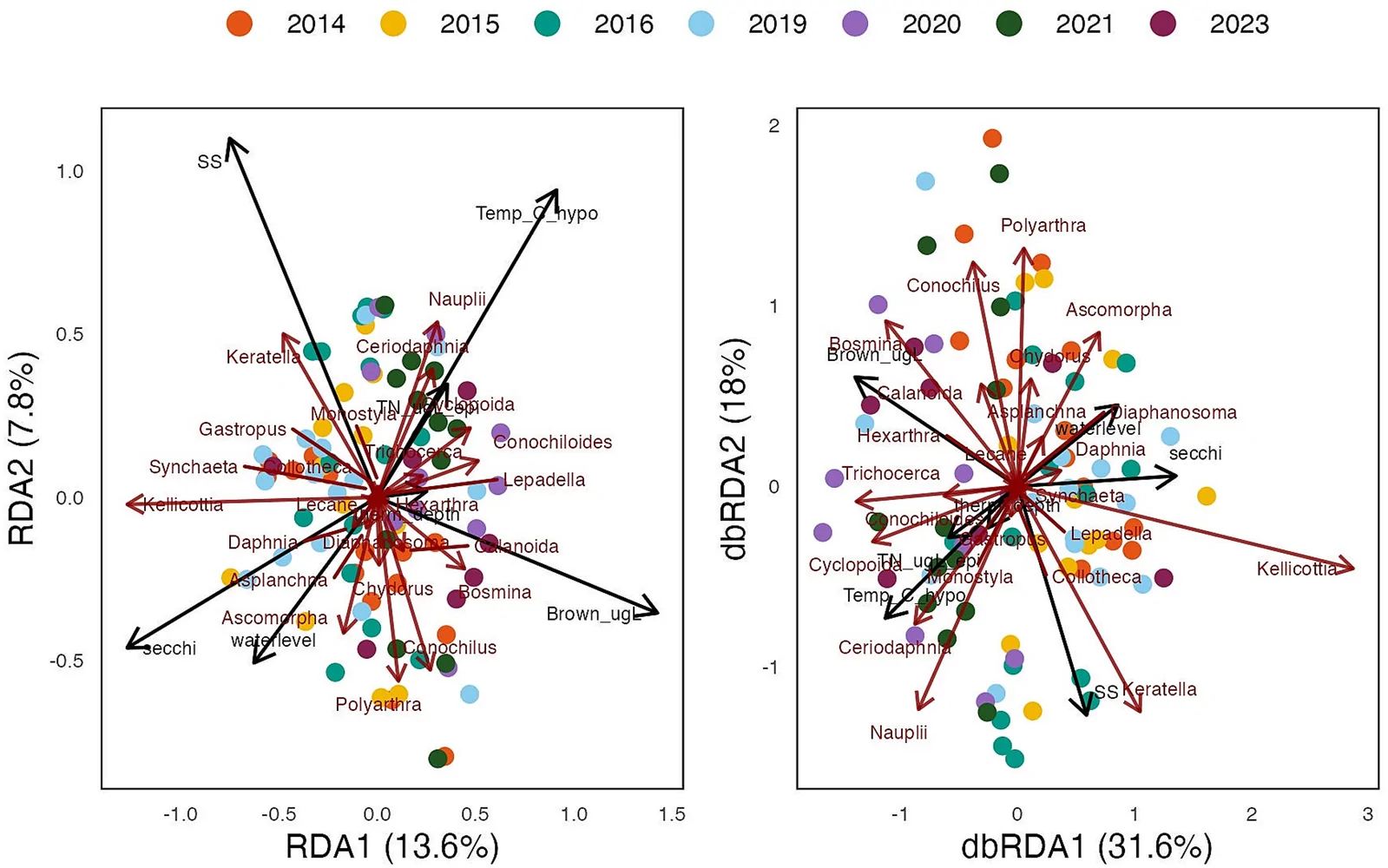
Redundancy analysis (RDA) of zooplankton community composition constrained by environmental variables in Beaverdam Reservoir. Samples (points) are colored by year to highlight temporal variation in community structure. Environmental variables (black arrows) indicate the direction and strength of relationships with the RDA axes. Only statistically significant drivers from permutation tests (*P* < 0.05) are shown. Species scores are indicated by red arrows. The proportion of constrained variance explained by each axis is reported in parentheses. SS = Schmidt stability, TP_ugL_epi = epilimnetic total phosphorus concentration, TN_ugL_epi = epilimnetic total nitrogen concentration, TN_ugL_hypo = hypolimnetic total nitrogen concentration, Temp_C_hypo = hypolimnetic water temperature, Brown_ugL = brown-algae pigments, DO_mgL_epi = epilimnetic dissolved oxygen concentration. Percentages on each axis indicate the unadjusted proportion of total variance in zooplankton community composition explained by that axis alone; the model’s overall explanatory power is reported as the adjusted *R*^2^ in the *Results* and should not equal the sum of the two plotted axes. Because marginal tests credit each variable only with variance explained independently of all other variables, percentages sum to less than the model’s total explained variance.

### Taxon-specific analyses identify temporal associations for key zooplankton taxa

Both ISA and SIMPER analyses identified a consistent set of taxa driving annual variation in zooplankton community composition. *Ascomorpha*, *Bosmina*, *Ceriodaphnia*, *Chydorus*, *Conochiloides*, *Gastropus*, and *Trichocerca* were identified as significant indicator taxa and major contributors to Bray–Curtis dissimilarity across pairwise year comparisons, suggesting that these taxa are the primary drivers of interannual community change in BVR (Fig. 5).

**Fig. 5:**
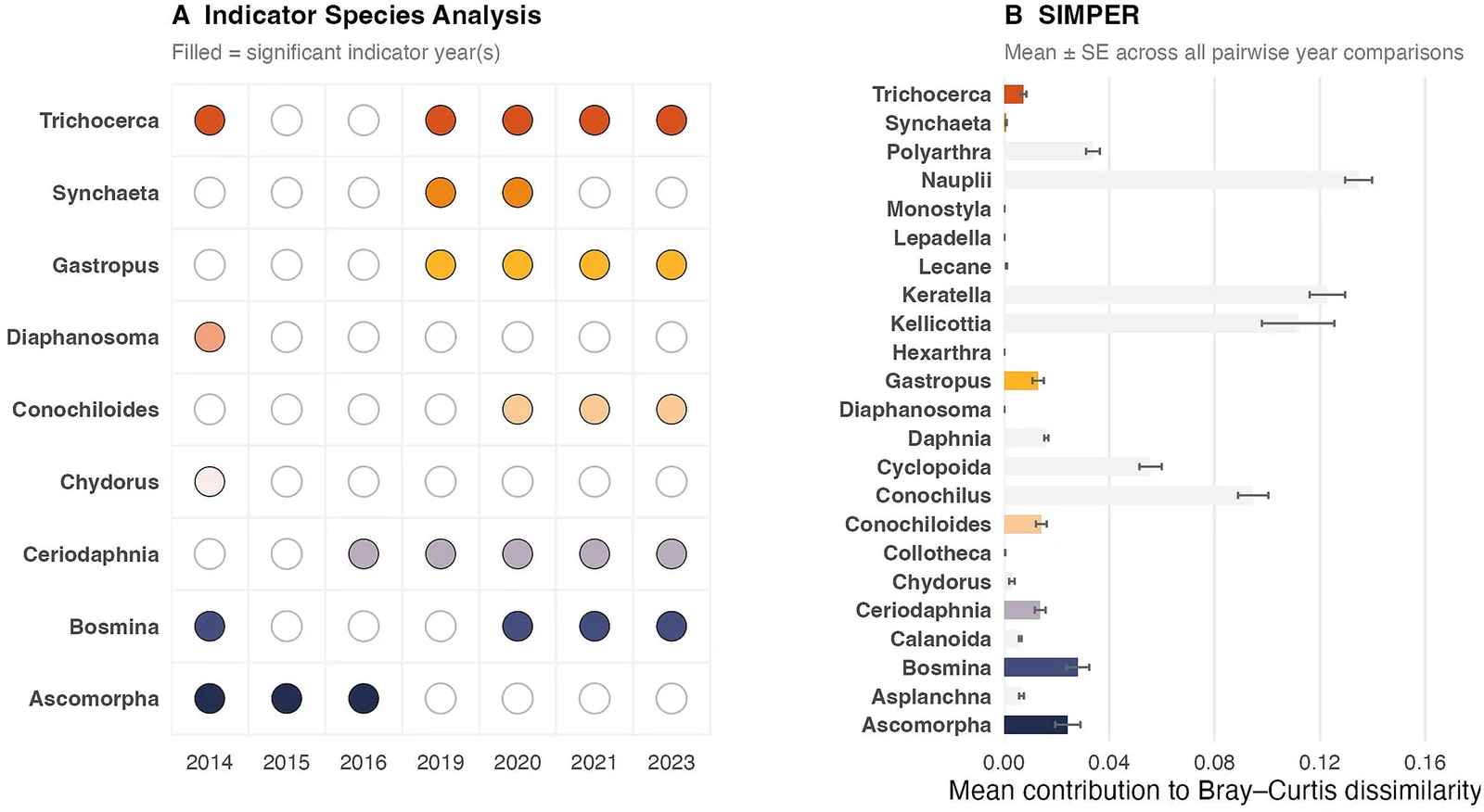
Comparison of taxa identified by indicator species analysis (ISA) and similarity percentage analysis (SIMPER) for interannual variation in zooplankton community composition. (A) ISA results showing significant indicator taxa and their associated years. Filled circles indicate years for which a taxon was identified as a significant indicator, whereas open circles indicate years with no significant association. (B) SIMPER results showing the mean (±SE) contribution of each taxon to Bray–Curtis dissimilarity across all pairwise year comparisons. Colored taxa were identified by both ISA and SIMPER, whereas gray taxa were identified only by ISA or SIMPER alone.

ISA identified nine taxa as significant indicators of specific year(s) (Fig. 5A, Table S8). Taxa fell into three broad groupings: those associated with early sampling years (2014–2016), those associated with later years (2019–2023), and those with associations spanning non-consecutive years. Early-year indicators (2014–2016) included *Ascomorpha, Diaphanosoma*, and *Chydorus*. Later-year indicators (2019–2023) were *Conochiloides*, *Gastropus*, and *Synchaeta*, reflecting a shift in community composition in more recent years. *Bosmina*, *Ceriodaphnia*, and *Trichocerca* showed associations across both early and late years.

SIMPER analysis revealed that copepod nauplii, *Keratella*, and *Kellicottia* were the largest mean contributors to Bray–Curtis dissimilarity across all pairwise year comparisons (Fig. 5B). Copepod nauplii mean contribution to Bray–Curtis dissimilarity was 0.13 ± 0.005 (±1 SE) to dissimilarity, followed by *Keratella* (0.12 ± 0.006) and *Kellicottia* (0.11 ± 0.013). Contributions of individual taxa to dissimilarity varied among year pairs, as shown in the supplement (Fig. S7).

## DISCUSSION

We used several multivariate approaches to identify the drivers of and changes to zooplankton community dynamics across temporal scales. We found that taxon-specific responses were likely related to environmental variability, particularly physical and chemical conditions. Our applications of first- and second-stage NMDS provided complementary information on annual and intra-annual zooplankton community dynamics. Additionally, RDA and dbRDA identified similar important environmental variables that were associated with zooplankton community structure. Lastly, ISA provided insight into which taxa were associated with specific years in our dataset, whereas SIMPER identified taxonomic signals over the full dataset. Below, we discuss our findings and consider how these methods can be used to identify ecological mechanisms driving zooplankton community variability in a eutrophic reservoir.

### Temporal patterns of zooplankton community structure

Within years, we observed seasonal patterns in the timing of zooplankton community density peaks that were broadly consistent with expected successional patterns described by the PEG model (Sommer *et al.*, 1986). Early in the season, food is not yet limiting and predation pressure on rotifers is comparatively low, allowing large, efficient grazers like *Daphnia* to persist alongside spiny and colonial rotifers before predation pressure intensifies later in the season (e.g. Allan, 1976; Sommer *et al.*, 1986; Marshall *et al.*, 2024). As the season progressed, nauplii density peaks preceded those of Cyclopoida, suggesting a recruitment lag in which nauplii mature into adults (e.g. Vanderploeg *et al.*, 2012). The shift in the BVR rotifer community composition from spiny (*Kellicottia*, *Keratella*) and colonial (*Conochilus*) rotifers to the evasive-swimming *Polyarthra* may be attributed to changes in seasonal predation pressure (e.g. *Cbaoborus*) or stratification dynamics (e.g. Stemberger and Evans, 1984; Hairston Jr. *et al.*, 2000). However, the lack of consistent invertebrate density data throughout the study period warrants further investigation of drivers of zooplankton community turnover in BVR. Furthermore, the later seasonal peak of *Bosmina* relative to *Ceriodaphnia* may reflect differences in life-history strategies, seasonal responses to resource availability, and competition, which have been shown to produce temporally distinct population maxima among these genera in lakes (Greenwood *et al.*, 1999; Gyllström, 2004). We note that our sampling from April to November excluded winter and early spring, which are biologically active months that would likely provide further insight to seasonal zooplankton community succession (Salonen *et al.*, 2009).

Across the 7-year study, zooplankton community composition shifted from being characterized by small cladocerans toward greater representation of rotifers. The shift in the importance of zooplankton taxa throughout our dataset, from *Chydorus* and *Ascomorpha* in earlier years to *Conochiloides*, *Gastropus*, and *Synchaeta* in later years, suggests that temporal variability identified by NMDS involved the replacement of taxa with distinct functional roles. Similar shifts in rotifer and cladoceran dominance have been documented in temperate lakes undergoing changes in trophic state, resource availability or seasonal habitat structure (e.g. Molinero *et al.*, 2006; Barbiero *et al.*, 2009; Arfè *et al.*, 2019). In contrast, *Bosmina*, *Ceriodaphnia*, and *Trichocerca* were associated with non-consecutive years across our study period, indicating that some taxa contributed to community variation through episodic fluctuations rather than consistent directional change. These findings suggest that interannual dynamics reflect not just species replacement but a shift in the range of feeding strategies represented in the community. Because the taxa contributing most strongly to temporal variation differ functionally and include both filter-feeding cladocerans and rotifers with more diverse feeding strategies (e.g. filter feeders, predators; Galir Balkić *et al.*, 2018), this turnover reflects a shift in functional, as well as taxonomic, structure rather than replacement among functionally similar taxa (Barnett *et al.*, 2007). Future studies that use complementary multivariate methods could shed more insight into these patterns for zooplankton.

Interestingly, zooplankton community composition exhibited interannual variability structured around a limited number of taxa that strongly characterized particular years. ISA identified three taxa associated with early years (2014–2016), three taxa associated with later years (2019–2023), and three taxa that did not show an association with either period. This result suggests that interannual variability reflects differences in temporal fidelity among taxa, with some taxa showing clearer association with particular years versus others. Similar patterns have been reported in long-term studies, where zooplankton communities often exhibit substantial interannual variability without clear directional change in overall composition, reflecting fluctuating contributions of taxa that respond differently to short-term environmental and biological variability (Adrian *et al.*, 2006; Jeppesen *et al.*, 2011). Within this framework, indicator taxa represent zooplankton community members with stronger temporal structuring in their occurrence or abundance, whereas non-indicator taxa may be more random or consistent within the community.

### Zooplankton responses to environmental drivers

Across analyses, zooplankton community structure was associated with both physical and chemical environmental drivers. Nutrient concentrations, Secchi depth, and phytoplankton biomass were consistent predictors of zooplankton community change, which has been supported by many other studies (Pinto-Coelhom *et al.*, 2005; Choi *et al.*, 2025; Yin *et al.*, 2025). Several physical metrics (e.g. Schmidt stability, thermocline depth) were also significant, suggesting that stratification may mediate shifts in zooplankton community structure by affecting vertical habitat availability, prey distribution, and predator–prey overlap (Gauthier *et al.*, 2014; Wang *et al.*, 2024). When comparing the relative importance of physical and chemical variables, we found that physical variables contributed slightly more to overall variation in zooplankton community structure, suggesting that mixing dynamics and the light environment were particularly important drivers of temporal zooplankton community dynamics, as has been found in other productive lakes (Weithoff *et al.*, 2000; van Gremberghe *et al.*, 2008; Toruan *et al.*, 2023).

The taxon-specific associations with environmental conditions can be explained by several ecological mechanisms. *Kellicottia* was positively associated with greater Secchi depth, consistent with a tolerance for low-nutrient, oligotrophic conditions (Stemberger and Gilbert, 1985). Conversely, brown-algae pigments were negatively associated with *Kellicottia*, suggesting that shifts in phytoplankton community composition may have reduced the availability of food resources preferred by this selective filter-feeding rotifer (Gilbert, 2022). *Keratella* was positively associated with Schmidt stability, potentially reflecting the strong seasonal structuring of this rotifer linked to water temperature (Stemberger and Evans, 1984). In contrast, *Polyarthra* and *Bosmina* were associated with weaker stratification strength (lower Schmidt stability) and lower water clarity, conditions typical of productive, frequently mixed systems where these taxa are often abundant (e.g. Sługocki and Czerniawski, 2018; Vucic, 2019). However, taxon-specific responses diverge considerably and species or morphotypes in broad zooplankton groups may not all respond in the same way. For example, *Keratella cochlearis* exhibits high morphological and genetic diversity, suggesting potential variation among morphotypes in responses to environmental conditions, including temperature (Cieplinski *et al.*, 2017). Overall, zooplankton community composition appeared to reflect the combined influence of physical and chemical conditions, with taxon-specific responses reflecting adaptations to different nutrient and stratification regimes.

### Utility of multiple multivariate approaches

The use of both first- and second-stage NMDS provided insight not just into temporal patterns but also into the ecological processes structuring zooplankton communities. For example, first-stage NMDS results indicated high variability in zooplankton community structure across years and months, as seen by the overlapping polygons (Fig. 2). However, centroids were somewhat spatially separated in ordination space, suggesting that there are some differences across years and months that may be driven by differences in environmental variables. Second-stage NMDS answered a slightly different question: are intra-annual zooplankton community dynamics similar across years? This analysis identified a signal that revealed an underlying pattern that was difficult to discern from solely looking at patterns in density among taxa. Several physical variables relating to thermal dynamics and water column mixing were associated with intra-annual patterns in zooplankton community composition, particularly in 2014 and 2019 (Fig. S6).

RDA and dbRDA complemented these unconstrained ordinations by quantifying the proportion of community variation explained by measured environmental variables. Both constrained ordinations identified similar environmental variables as the primary drivers of zooplankton community composition, suggesting that linear methods adequately captured the community–environment relationships in this dataset. While direct comparisons of RDA and dbRDA in lake zooplankton are rare, theoretical considerations indicate that linear and distance-based constrained methods often produce similar ordination patterns when community composition changes relatively little along environmental gradients (Anderson, 2001). Therefore, we expect that datasets encompassing greater taxonomic diversity, stronger compositional turnover, or broader environmental gradients would benefit more from comparing RDA and dbRDA.

Pairing ISA and SIMPER allowed us to distinguish two different lines of evidence for community change that either method alone would have conflated: a taxon’s temporal specificity and its contribution to compositional dissimilarity. Taxa flagged by both approaches are the strongest candidates as drivers of interannual variation, having satisfied both of these independent criteria. The two analyses did not fully align, however, and that divergence is itself informative. Taxa contributing substantially to dissimilarity, but not flagged as significant indicators, likely reflect broadly abundant taxa whose shifts were large in magnitude but not tied to a specific year. Conversely, indicator taxa with smaller dissimilarity contributions highlight species that are associated with particular years rather than those that numerically dominate community-wide change. Relying on SIMPER alone would have emphasized abundance-driven shifts at the expense of less common but temporally diagnostic taxa, while relying on ISA alone would have understated how much dominant taxa shaped overall compositional turnover. Together, the two approaches give a more holistic picture of interannual variation in this community, capturing both which taxa changed the most and which changed in ways most diagnostic of specific years.

### Potential multivariate analysis decision workflow

Our primary objective was not to evaluate individual multivariate methods but to demonstrate how multiple commonly used methods can be applied together in a complementary manner to provide more biologically interpretable insights into temporal zooplankton community dynamics. Throughout this study, unconstrained ordination, constrained ordination, and taxon-specific analyses each addressed distinct but interconnected ecological questions, linking community structure change to individual taxa and their environmental drivers.

The conceptual framework presented in Fig. 6 generalizes this integrative approach by placing the analyses used here within the broader suite of multivariate methods commonly applied in community ecology. We note that this framework is not meant to be a comprehensive list of all multivariate resources available to zooplankton researchers. Instead, this framework is intended to serve as a guide for using several common methods, emphasizing how study objectives should guide method selection and how complementary analyses may be combined to address different levels of ecological organization. For example, exploratory ordinations are well suited for visualizing patterns in community composition, constrained ordinations quantify relationships with measured environmental gradients, and taxon-specific analyses identify the relevant taxa driving observed patterns. Additional approaches, including partial ordination and variation partitioning, extend this framework by allowing researchers to separate the contributions of environmental, spatial, and temporal processes when these factors are of interest (Fig. 6).

**Fig. 6:**
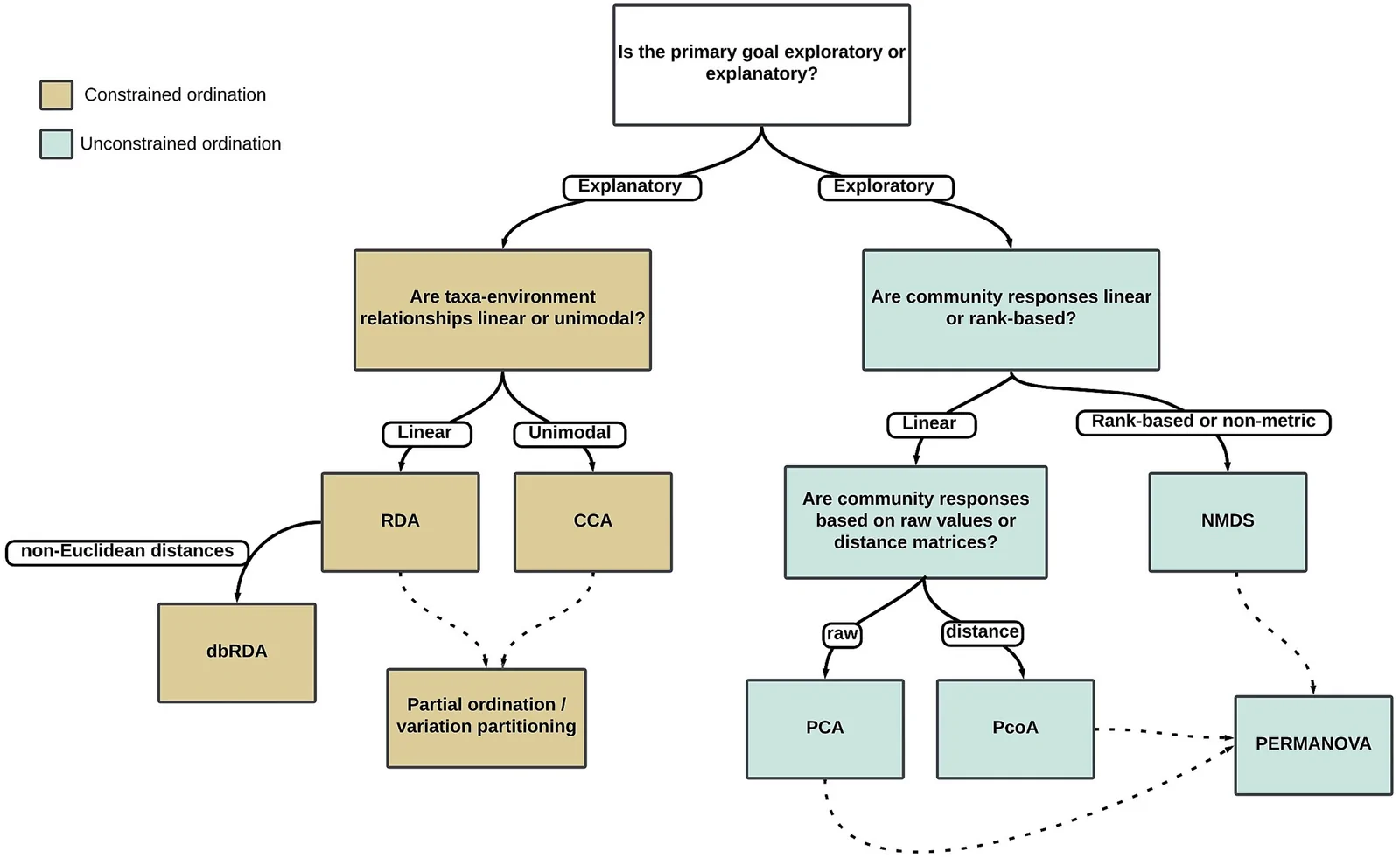
Conceptual workflow for selecting multivariate approaches to analyze ecological community datasets. The left branch (explanatory analyses) guides selection among redundancy analysis (RDA) and canonical correspondence analysis (CCA), depending on whether taxon responses to environmental variables are linear (RDA; shown in this study) or unimodal (CCA; not applied because of the linear structure of our data). The right branch (exploratory analyses) guides selection among principal coordinates analysis (PCoA) and non-metric multidimensional scaling (NMDS), depending on whether relationships are linear or distance based, respectively. Partial ordination (partial RDA or partial CCA) and permutational multivariate analysis of variance (PERMANOVA) are shown with dotted lines to indicate optional complementary analyses (not part of the decision pathway) that can be used alongside ordinations to partition variation or test for group differences, rather than as sequential steps in the decision tree. Indicator species analysis (ISA) and SIMPER are not included as decision nodes, as they do not guide ordination selection; instead, these methods may be applied as complementary, taxon-focused analyses to identify taxa driving the differences among groups that emerged from the ordination results. Environmental variables may be overlaid onto unconstrained ordinations (right branch) to assess *post hoc* correlations with community metrics.

Although the specific analytical pathway will depend on the dataset and research question, integrating complementary multivariate approaches can strengthen ecological inference by linking patterns, potential drivers, and contributing taxa within a unified analytical framework. We anticipate that this conceptual workflow may be particularly useful for long-term plankton monitoring studies, where combining multiple methods can yield more robust and biologically meaningful interpretations of community change, especially given the sparse nature of zooplankton datasets, with differing levels of taxonomic aggregation.

### Considerations for scaling analyses

We compared RDA and dbRDA using the same zooplankton dataset to identify whether these similar methods would yield different results. Ultimately, we found that RDA and dbRDA were comparable in our study; however, we expect this convergence to break down in systems with greater taxonomic diversity or stronger zero-inflation than ours. For example, datasets spanning broader spatial or temporal scales, or community matrices with many infrequent or sporadically occurring taxa, may better highlight differences between these two methods, as double zeros could inflate Euclidean distances (RDA) in ways that Bray–Curtis distances (dbRDA) discount, potentially leading the two approaches to identify different dominant gradients or environmental drivers. In our case, both dbRDA and RDA identified a largely overlapping set of significant environmental variables, pointing to water column structure, stratification, and nutrient availability as the primary drivers of community composition. This convergence likely reflects the relative compositional stability of BVR during the study period, in the absence of a major disturbance or shift in dominant taxa.

The other approaches used in this study were similarly scalable. Standard NMDS visualized overall patterns of community dissimilarity among sampling events, while second-stage NMDS evaluated whether intra-annual patterns in community structure were consistent across years. Additionally, SIMPER and ISA identified which specific taxa contributed most to compositional differences among years, providing taxon-level detail that the other approaches did not capture. However, a longer time series may provision second-stage NMDS with greater power to detect consistent intra-annual patterns or enable ISA and SIMPER to identify different key taxa following compositional shifts that may occur in longer timeseries. Together, these approaches offered insight into the structure of community variation (RDA/dbRDA), its temporal repeatability (NMDS/second-stage NMDS), and the specific taxa driving observed differences (SIMPER/ISA).

More broadly, the transferability of our findings depends on plankton dataset characteristics that vary considerably across systems. Researchers working with longer time series or more heterogeneous communities may find that the choice between multivariate methods has greater consequences for ecological inference than observed here. We therefore encourage researchers to consider how zero-inflation, dataset length, and the stability of community composition in their own systems may influence the relative utility of these approaches, reinforcing the value of applying multiple complementary methods when community composition is more variable or diverse.

## CONCLUSION

Across the 7-year study period, we demonstrate how commonly used multivariate methods can be applied in a complementary manner for studying zooplankton community dynamics within and across years. We found that zooplankton communities were highly variable across years and months, and that physical and chemical variables relating to stratification were important drivers of zooplankton community structure over the 7-year period. Specifically, several cladoceran and rotifer taxa were found to be associated with in-reservoir stratification conditions. By integrating unconstrained ordination, constrained ordination, and taxon-specific analyses, we show that these methods provide complementary perspectives on community dynamics, linking broad patterns of temporal variation to both environmental structure and the taxa most responsible for compositional change. This multi-method approach highlights that no single multivariate technique fully captures the complexity of ecological communities in isolation. Therefore, our study provides a robust and biologically interpretable basis for understanding zooplankton community dynamics. We envision that this framework can be applied to other long-term ecological datasets where multiple processes shape temporal variability.

## Supplementary Material

Supplementary_files_fbag082

## Data Availability

All datasets analyzed in this study are publicly available through the Ecological Data Initiative (EDI) repository (Carey et al., 2022b; Carey *et al.*, 2024a; Carey *et al.*, 2024b; Carey *et al.*, 2024c; Carey *et al.*, 2024d; Carey *et al.*, 2024e; Carey *et al.*, 2026). The code used to generate all analyses and statistics is available in the Zenodo repository (Wander *et al.*, 2026).

## Notes

Corresponding Editor: Lars-Anders Hansson
