# Supplementary material for: A multivariate analysis framework for interpreting long-term zooplankton community dynamics
Source: J Plankton Res. 2026 Oct 30;48(6):fbag082. doi: 10.1093/plankt/fbag082 (PMC13720733; doi:10.1093/plankt/fbag082)
Supplement: Supplementary file 1 [file supplementary_files_fbag082.zip › SI_interannual_zoops_25Sep2026_fbag082.pdf]

Supplement to: A multivariate analysis framework for interpreting long-term zooplankton community dynamics

Heather L. Wander[1]*, Mary E. Lofton[1], Jonathan P. Doubek[2,3], Bryan L. Brown[1], Cayelan C. Carey[1]

[1] Department of Biological Sciences, Virginia Tech, Blacksburg, Virginia, USA

[2] School of Natural Resources, Lake Superior State University, Sault Ste. Marie, Michigan USA

[3] Center for Freshwater Research and Education, Lake Superior State University, Sault Ste. Marie, Michigan, USA

*Corresponding author: hwander@vt.edu

Submitted to Journal of Plankton Research

Number of Tables: 8

Number of Figures: 7

Table S1: Summary statistics (minimum, maximum, and percent missing) for environmental variables collected from Beaverdam Reservoir during 2014-2023.

Environmental variable	Min.	Max.	% missing
Epilimnetic total nitrogen (µg/L)	143.10	2433	20.22
Hypolimnetic total nitrogen (µg/L)	45.0	453	19.10
Epilimnetic total phosphorus (µg/L)	4.55	56.10	20.22
Hypolimnetic total phosphorus (µg/L)	1.20	27.7	19.10
Epilimnetic water temperature (°C)	10.51	27.60	13.48
Hypolimnetic water temperature (°C)	8.42	17.08	13.48
Epilimnetic dissolved oxygen (µg/L)	3.69	11.84	14.61
Water level (m)	9.36	13.50	0
Thermocline depth (m)	0.55	8.95	13.48
Oxycline depth (m)	0.25	10.50	13.48
Schmidt stability (J/m^2)	0.09	137.11	8.99
Green algae pigments (µg/L)	0.91	14.24	35.96
Bluegreen algae pigments (µg/L)	0.01	12.19	35.96
Brown algae pigments (µg/L)	0.48	15.42	35.96
Mixed algae pigments (µg/L)	0	8.06	35.96
Secchi depth (m)	0.90	4.7	17.98
Air temperature (°C)	1.62	30.09	0
Shortwave radiation (W/m^2)	59.41	338.36	0
Longwave radiation (W/m^2)	213.24	421.82	0
Relative humidity (%)	44.29	97.88	0
Wind speed (m/s)	1.32	8.21	0
Rain (m/day)	0	0.05	0

Table S2: Summary of the 23 zooplankton taxa identified in BVR over the seven-year study period, including the frequency of occurrence and percent contribution of each taxon to total community density. Frequency is the percentage of sampling dates for which a taxon was observed. Percent (%) contribution to total density was calculated by summing average densities for each taxon by date across the full study period and dividing by the summed density of all zooplankton taxa. Taxa are listed in descending order of percent contribution.

Taxon	Frequency	% contribution
Nauplii	98.9	26.18
Kellicottia	78.7	21.83
Keratella	97.8	16.30
Conochilus	65.2	11.00
Cyclopoida	98.9	8.13
Polyarthra	75.3	3.87
Ascomorpha	33.7	2.18
Bosmina	48.3	1.98
Daphnia	84.3	1.98
Gastropus	33.7	1.90
Ceriodaphnia	40.4	1.00
Conochiloides	27	0.97
Trichocerca	40.4	0.82
Calanoida	30.3	0.65
Asplanchna	15.7	0.59
Synchaeta	12.4	0.25
Chydorus	15.7	0.15
Collotheca	7.9	0.08
Lecane	10.1	0.07
Monostyla	4.5	0.03
Lepadella	2.2	0.02
Diaphanosoma	5.6	0.01
Hexarthra	1.1	0

Table S3: Environmental driver loadings from *envfit* analysis, including vector scores along first-stage NMDS axes 1, 2, and 3, and permutation-based p-values (999 permutations). Significant p-values are shown in bold.

Environmental variable	NMDS1	NMDS2	NMDS3	P-value
Green algae pigments (μg/L)	-0.1667	0.1674	-0.3412	**0.001**
Bluegreen algae pigments (μg/L)	-0.2186	0.0898	-0.3804	**0.001**
Brown algae pigments (μg/L)	-0.0920	-0.2931	-0.4376	**0.001**
Secchi depth (m)	0.2741	0.0050	0.4897	**0.001**
Epilimnetic dissolved oxygen (μg/L)	-0.0067	-0.149	0.4032	**0.002**
Hypolimnetic water temperature (°C)	-0.1349	0.0197	-0.3601	**0.004**
Hypolimnetic total nitrogen (μg/L)	-0.2034	0.0306	-0.3121	**0.007**
Hypolimnetic total phosphorus (μg/L)	-0.1721	-0.0481	-0.3275	**0.007**
Schmidt stability (J/m^2)	-0.1463	0.2480	0.1774	**0.018**
Mixed algae pigments (μg/L)	0.0099	0.2716	-0.2065	**0.020**
Epilimnetic water temperature (°C)	-0.2297	0.2037	0.0328	**0.043**
Oxycline depth (m)	0.0833	-0.0698	0.2713	0.080
Longwave radiation (W/m^2)	-0.1344	0.2187	0.0668	0.102
Epilimnetic total phosphorus (μg/L)	0.0441	0.2033	0.1590	0.116
Air temperature (°C)	-0.0709	0.2394	0.0201	0.154
Water level (m)	0.0041	-0.0149	0.2221	0.253
Wind speed (m/s)	-0.1165	-0.1794	0.0517	0.270
Epilimnetic total nitrogen (μg/L)	-0.0649	0.0323	-0.1884	0.357
Rain (m/day)	0.0048	0.1113	-0.1063	0.579
Thermocline depth (m)	0.0840	0.0118	-0.1176	0.639
Relative humidity (%)	0.0509	0.0661	0.0159	0.901

Table S4: Environmental driver loadings from *envfit* analysis, including vector scores along second-stage NMDS axes 1, 2, and 3, and permutation-based p-values (999 permutations). Significant p-values are shown in bold.

Environmental variable	NMDS1	NMDS2	NMDS3	P-value
Water level (m)	-0.9116	-0.1406	0.3708	**0.001**
Schmidt stability (J/m^2)	-0.8350	0.3316	0.3992	**0.015**
Secchi depth (m)	-0.8882	0.3616	0.0613	0.053
Epilimnetic water temperature (°C)	-0.5016	0.7703	-0.1324	0.096
Hypolimnetic total phosphorus (µg/L)	0.7063	-0.5250	-0.2752	0.103
Brown algae pigments (µg/L)	0.8371	-0.1081	-0.1459	0.194
Air temperature (°C)	-0.6012	0.5707	0.2449	0.203
Epilimnetic total nitrogen (µg/L)	-0.0753	0.8260	0.0799	0.212
Hypolimnetic water temperature (°C)	0.7673	-0.023	-0.0345	0.343
Hypolimnetic total nitrogen (µg/L)	0.7425	-0.1249	-0.2858	0.354
Bluegreen algae pigments (µg/L)	0.5976	0.2968	0.3352	0.364
Wind speed (m/s)	-0.1614	0.5278	0.4325	0.493
Green algae pigments (µg/L)	0.2086	0.5472	0.3551	0.524
Longwave radiation (W/m^2)	-0.5422	0.2136	0.3392	0.547
Relative humidity (%)	-0.3001	-0.6039	-0.0935	0.550
Thermocline depth (m)	0.4512	-0.4687	-0.1247	0.573
Epilimnetic total phosphorus (µg/L)	-0.4344	0.0459	-0.4612	0.628
Mixed algae pigments (µg/L)	-0.4005	0.4546	-0.0125	0.688
Rain (m/day)	0.1646	-0.3386	-0.0801	0.872
Oxycline depth (m)	0.0161	-0.0690	-0.3323	0.935
Epilimnetic dissolved oxygen (µg/L)	-0.1973	0.2833	-0.1986	0.969

Table S5: Redundancy analysis (RDA) results of axis-level significance. F-values and p-values from PERMANOVA indicate which canonical axes significantly explain constrained variation in zooplankton community composition. Variance explained (%) refers to the proportion of constrained variation captured by each axis. Note that axes represent linear combinations of environmental variables that explain variation in zooplankton community composition. Significant p-values are shown in bold.

Axis	F-value	P-value	Variance explained (%)
1	14.91	**0.001**	13.6
2	8.52	**0.013**	7.8
3	6.50	0.088	5.9
4	4.50	0.633	4.1
5	2.99	0.963	2.7
6	2.19	1.000	2.0
7	1.27	NA	1.2
8	0.98	NA	0.9
9	0.92	NA	0.8
10	0.51	NA	0.5
11	0.42	NA	0.4
12	0.24	NA	0.2
13	0.16	NA	0.1
14	0.10	NA	0.1
15	0.07	NA	0.1
16	0.03	NA	0
17	0.01	NA	0
18	0.01	NA	0
19	0.01	NA	0

Table S6: Distance-based redundancy (dbRDA) results showing axis-level significance. F-values and p-values from PERMANOVA indicate which canonical axes significantly explain constrained variation in zooplankton community composition. Variance explained (%) refers to the proportion of constrained variation captured by each axis. Significant p-values are italicized. Note that axes represent linear combinations of environmental variables that explain variation in zooplankton community composition. Significant p-values are shown in bold.

Axis	F-value	P-value	Variance explained (%)
1	12.92	**0.001**	31.6
2	7.38	**0.005**	18.0
3	5.59	**0.021**	13.7
4	4.09	0.353	10.0
5	2.55	0.963	6.2
6	1.98	1.00	4.8
7	1.42	NA	3.5
8	1.13	NA	2.8
9	0.91	NA	2.2
10	0.65	NA	1.6
11	0.51	NA	1.3
12	0.46	NA	1.1
13	0.37	NA	0.9
14	0.31	NA	0.7
15	0.23	NA	0.6
16	0.14	NA	0.3
17	0.10	NA	0.2
18	0.08	NA	0.2
19	0.07	NA	0.2

Table S7: Variation partitioning results from RDA, showing the unique and shared contributions of physical and chemical variable groups to zooplankton community variance. Physical and chemical variables are the same as those used in the RDA and dbRDA analyses (Tables 1-2). Adj. R^2 values are shown for each fraction; [a] and [b] represent variance explained uniquely by physical and chemical variables respectively, [d] represents shared variance, and [h] represents residual (unexplained) variance. Note that meteorological variables (shortwave radiation, relative humidity, wind speed, rainfall) were not significant predictors of zooplankton community structure and are therefore excluded from the partitioning results.

Component	Adj R^2
Physical (unique)	0.084
Chemical (unique)	0.062
Meteorological (unique)	0.000
Physical + Chemical	0.056
Physical + Meteorological	-0.001
Chemical + Meteorological	-0.008
Physical + Chemical + Meteorological	0.04
Residual	0.768

Table S8: Results of Indicator Species Analysis (ISA) for zooplankton taxa across seven sampling years in Beaverdam Reservoir. IndVal is the indicator value statistic reflecting the association strength between a taxon and its indicated year(s). A is specificity (probability that the community belongs to the indicated year(s) given the taxon is present); B is fidelity (probability of finding the taxon in the indicated year(s)). Only taxa with significant p-values (< 0.05) are shown.

Taxon	Associated years	IndVal	A	B	p
Ascomorpha	2014, 2015, 2016	0.78	0.96	0.634	0.001
Bosmina	2014, 2020, 2021, 2023	0.718	0.822	0.628	0.023
Ceriodaphnia	2016, 2019, 2020, 2021, 2023	0.692	0.943	0.508	0.032
Chydorus	2014	0.627	0.786	0.5	0.003
Conochiloides	2020, 2021, 2023	0.753	0.913	0.621	0.001
Diaphanosoma	2014	0.459	0.737	0.286	0.023
Gastropus	2019, 2020, 2021, 2023	0.798	1	0.636	0.001
Synchaeta	2019, 2020	0.632	1	0.4	0.002
Trichocerca	2014, 2019, 2020, 2021, 2023	0.704	0.897	0.552	0.01

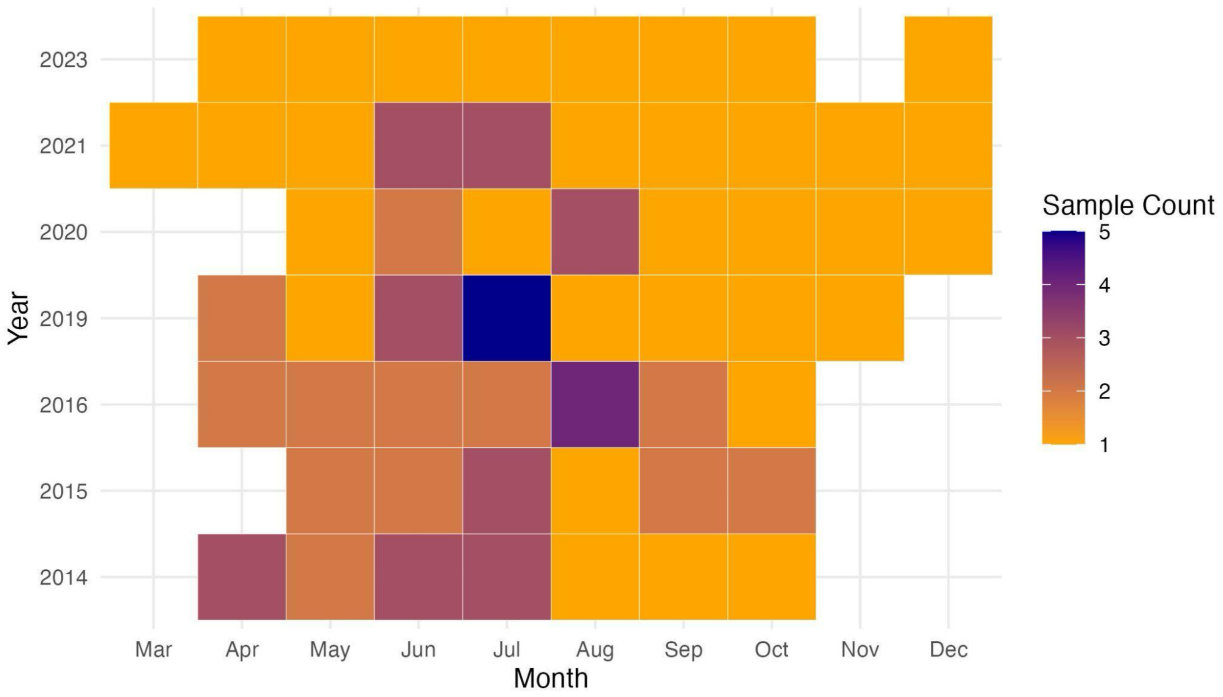

Figure S1: Temporal coverage of zooplankton samples in Beaverdam Reservoir from 2014-2023.

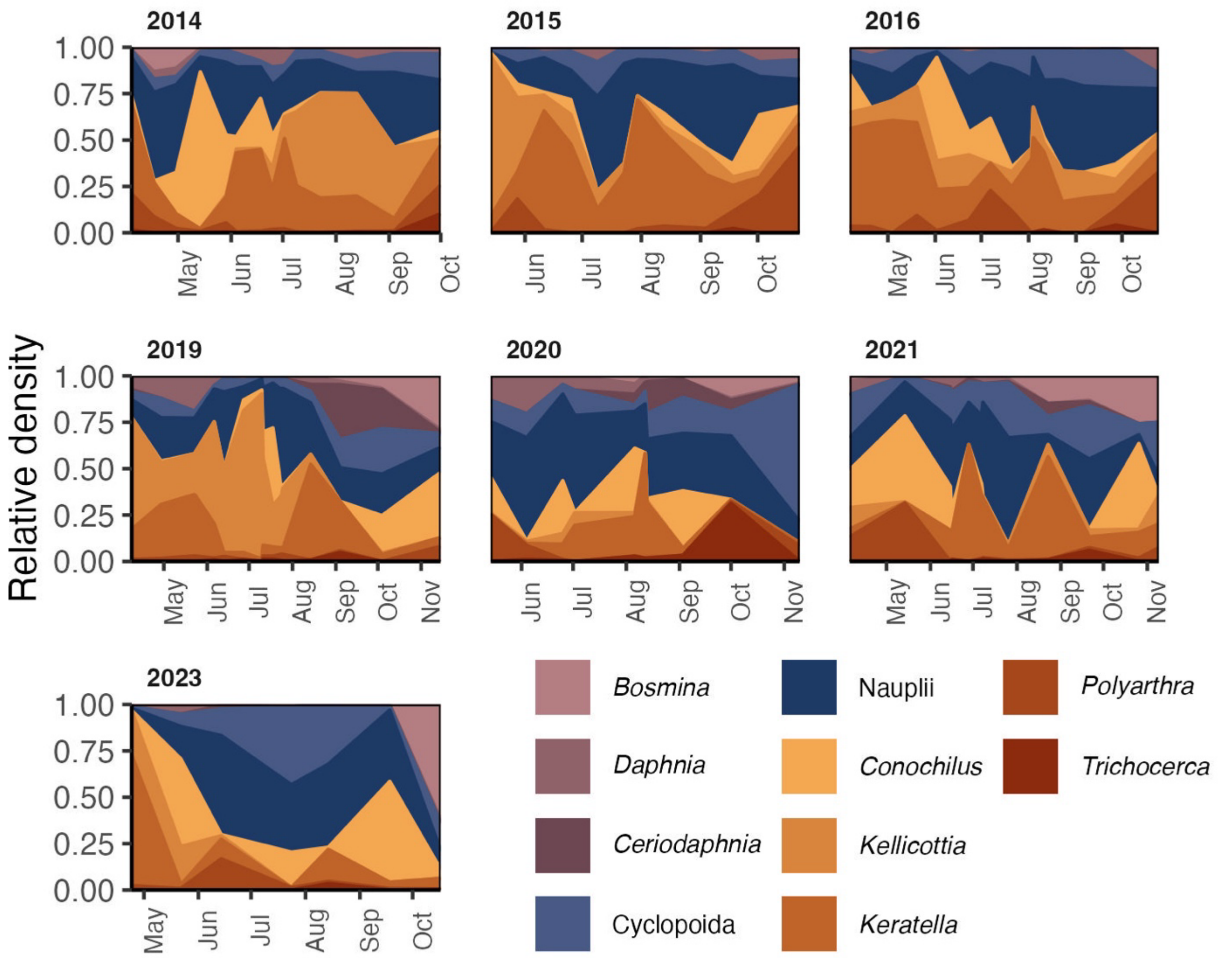

Figure S2: Relative zooplankton density for the ten common taxa observed in Beaverdam Reservoir from May-October 2014-2023. Panels depict each of the seven non-consecutive years included in this study. Cladocera are shown in mauve, Copepoda in blue, and Rotifera in orange/brown. Note different x-axis ranges corresponding to when the first and last zooplankton samples were taken each year.

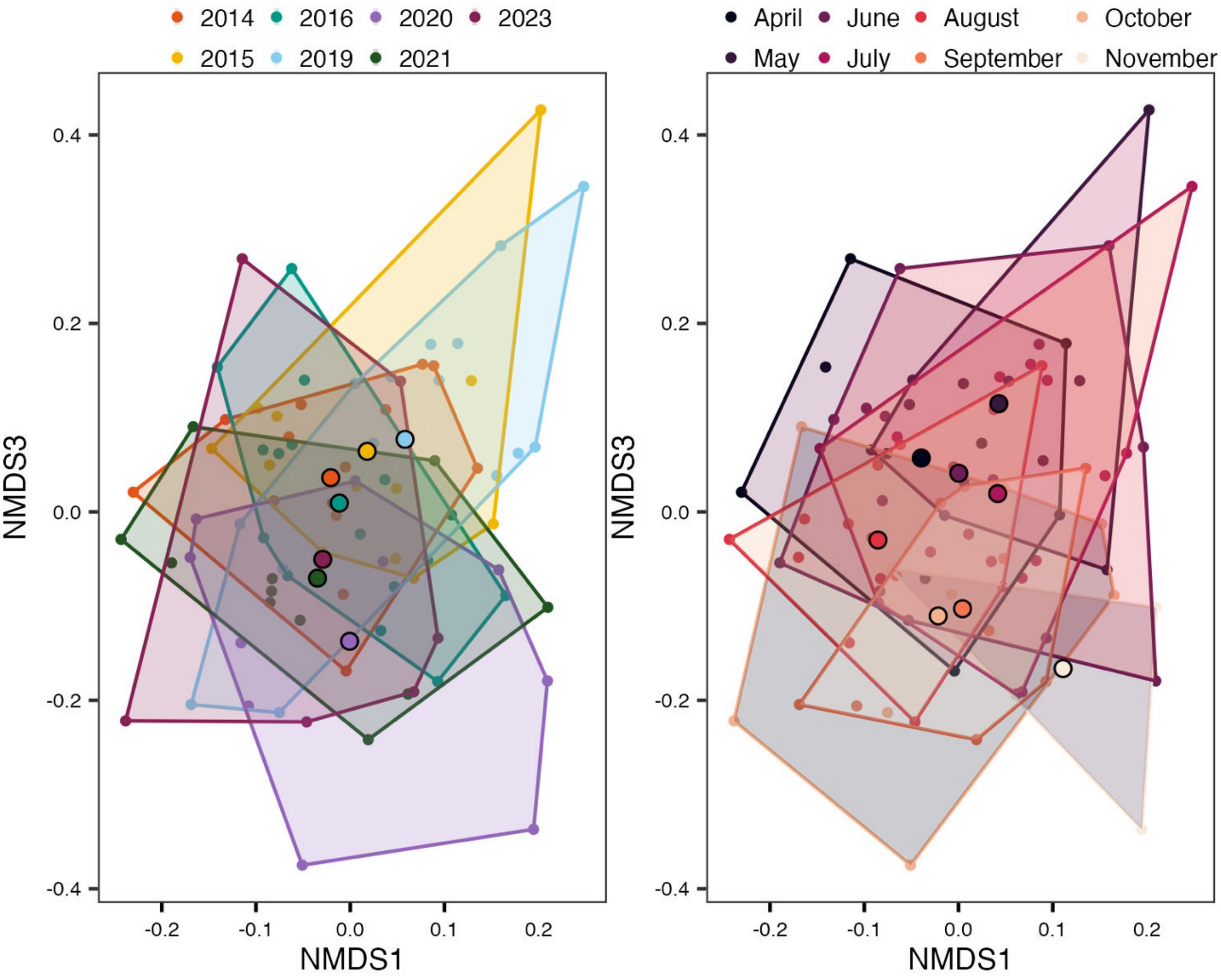

Figure S3: First-stage Non-metric MultiDimensional Scaling (NMDS) ordination for axes 3 versus 1 using zooplankton density data from Beaverdam Reservoir (k = 3; stress = 0.14). Both panels depict the same ordination with different polygon groupings, where points summarize temporal trends in summer community structure data from distinct sampling days. Large colored points represent the centroid of each polygon. Polygons in the left panel correspond to years (n = 6) and polygons in the right panel correspond to months (n = 8). This ordination summarizes zooplankton data collected from n = 85 distinct sampling days.

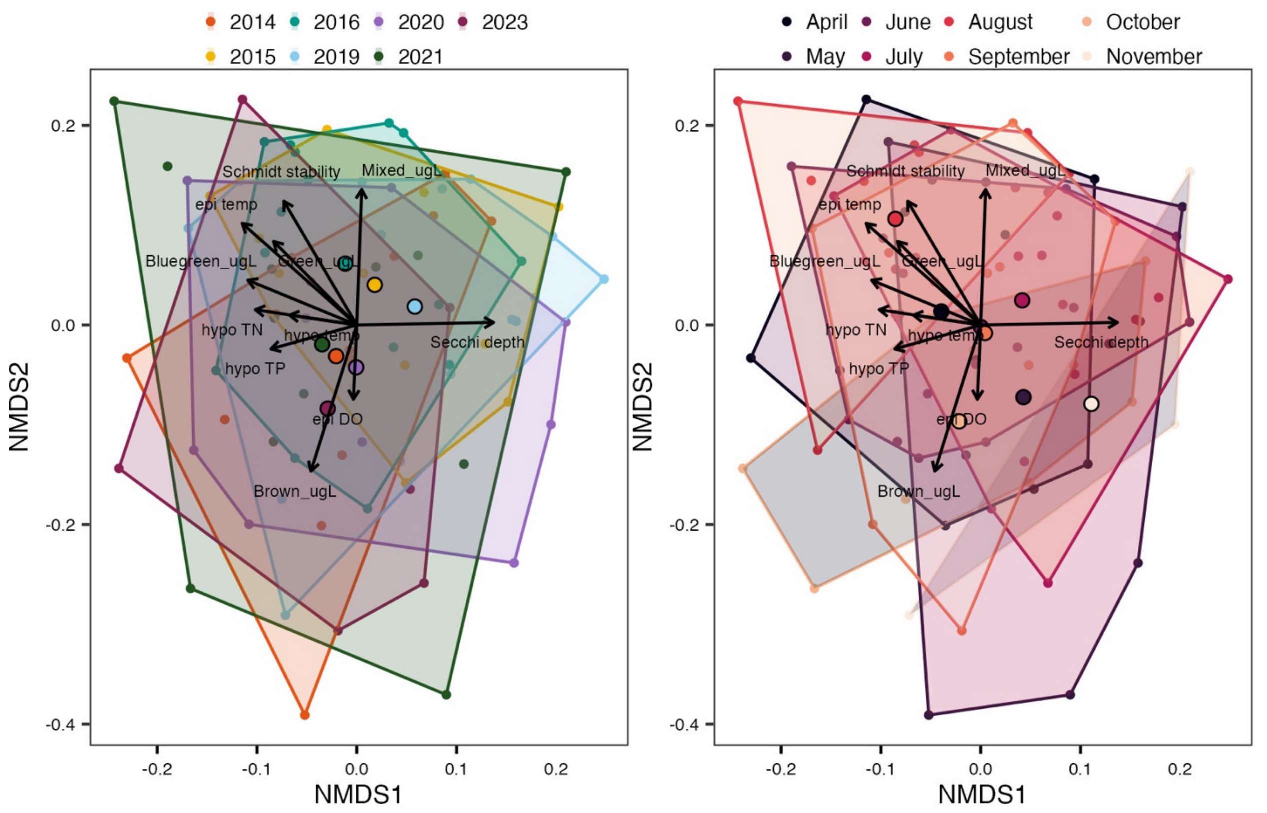

Figure S4: First-stage Non-metric MultiDimensional Scaling (NMDS) ordination for axes 2 versus 1 with significant environmental drivers overlaid as arrows (k = 3; stress = 0.14). Both panels depict the same ordination with different polygon groupings, where points summarize temporal trends in summer community structure data from distinct sampling days. Large colored points represent the centroid of each polygon. Polygons in the left panel correspond to years (n = 6) and polygons in the right panel correspond to months (n = 8). This ordination summarizes zooplankton data collected from n = 85 distinct sampling days. Bluegreen_ugL = Bluegreen (cyanobacteria) algae pigments; Green_ugL = Green algae pigments; Brown_ugL = Brown algae pigments, Mixed_ugL = Mixed algae pigments; epi temp = epilimentic water temperature; hypo temp = hypolimnetic water temperature; hypo TN = hypolimnetic total nitrogen concentration; hypo TP = hypolimnetic total phosphorus concentration; epi DO = epilimnetic dissolved oxygen concentration. See Table S3 for environmental variable units.

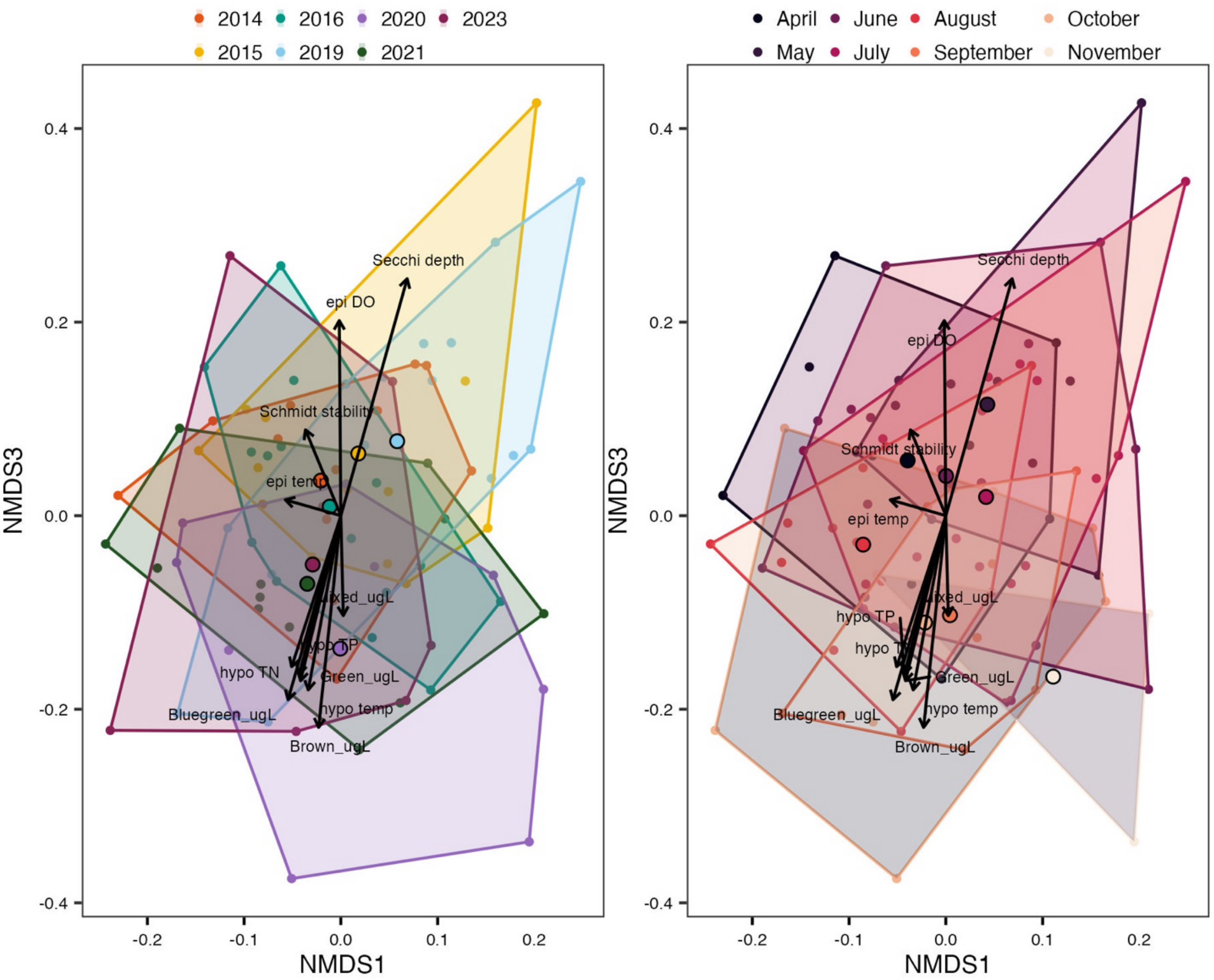

Figure S5: First-stage Non-metric MultiDimensional Scaling (NMDS) ordination for axes 3 versus 1 with significant environmental drivers overlaid as arrows (k = 3; stress = 0.14). Both panels depict the same ordination with different polygon groupings, where points summarize temporal trends in summer community structure data from distinct sampling days. Large colored points represent the centroid of each polygon. Polygons in the left panel correspond to years (n = 6) and polygons in the right panel correspond to months (n = 8). This ordination summarizes zooplankton data collected from n = 85 distinct sampling days. Bluegreen_ugL = Bluegreen (cyanobacteria) algae pigments; Green_ugL = Green algae pigments; Brown_ugL = Brown algae pigments, Mixed_ugL = Mixed algae pigments; epi temp = epilimentic water temperature; hypo temp = hypolimnetic water temperature; hypo TN = hypolimnetic total nitrogen concentration; hypo TP = hypolimnetic total phosphorus concentration; epi DO = epilimnetic dissolved oxygen concentration. See Table S3 for environmental variable units.

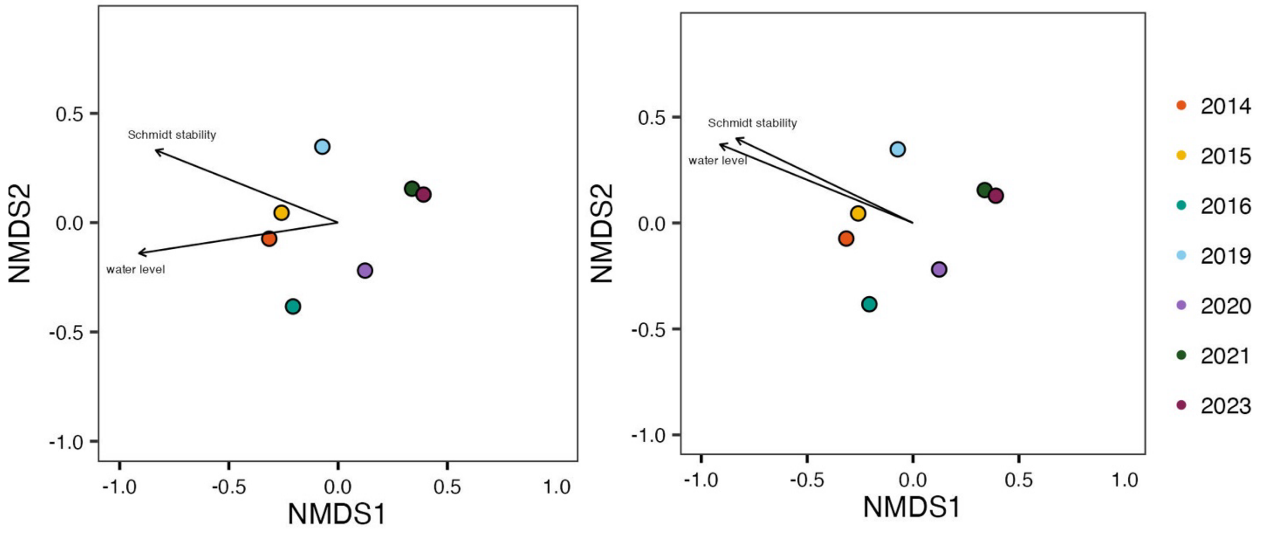

Figure S6: Second-stage Non-metric MultiDimensional Scaling (NMDS; k = 3, stress = 0.10) with significant environmental variables overlaid as arrows, calculated using pairwise correlations among all distance matrices for each year of zooplankton density data. Grouping of years (colors) indicate similarities in intra-annual patterns of zooplankton community structure in Beaverdam Reservoir. Left panel corresponds to axis 2 versus axis 1; right panel corresponds to axis 3 versus axis 1. Note that samples were aggregated to the monthly scale for May-October each year to meet the equal sample size requirement for this analysis.

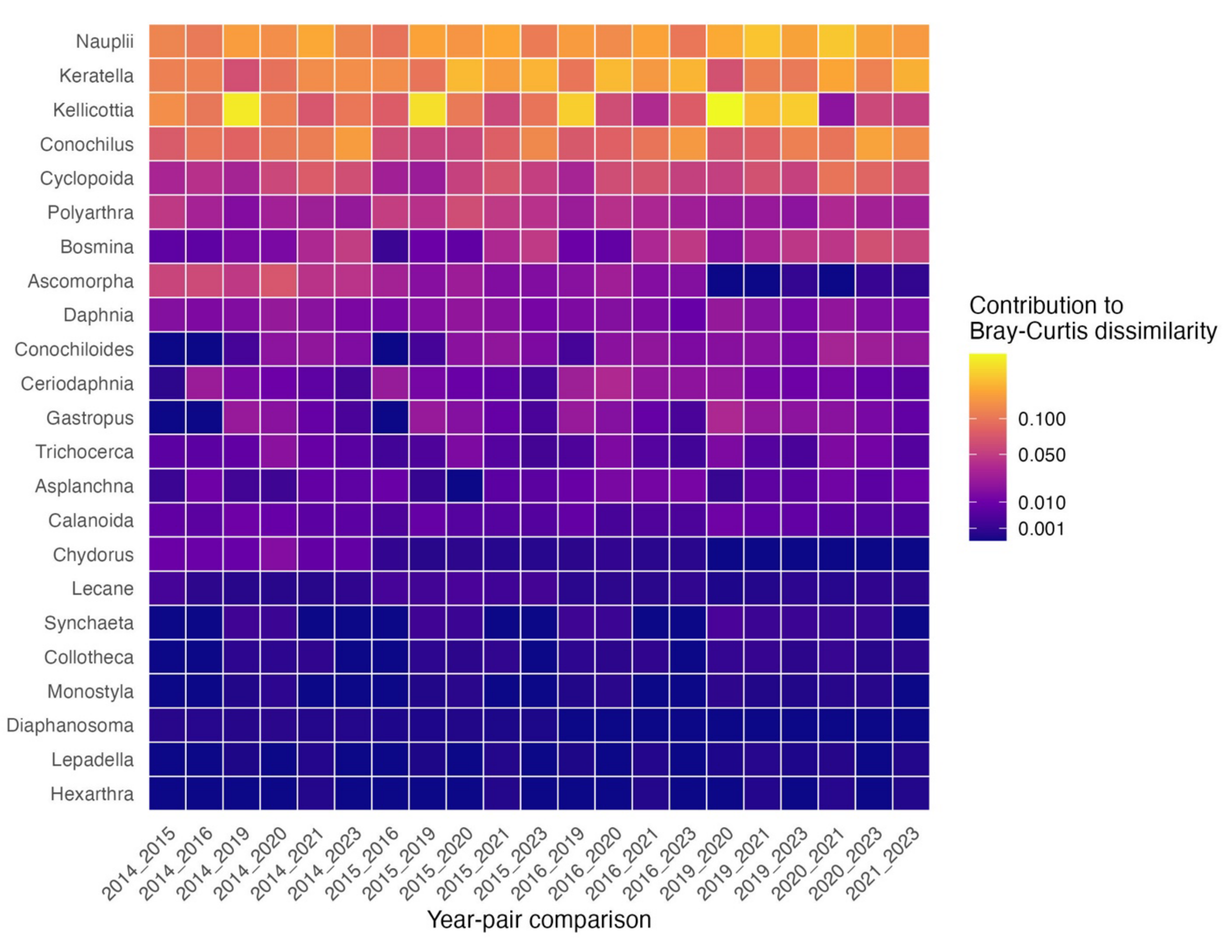

Figure S7: Heatmap of SIMPER contributions to Bray-Curtis dissimilarity for each taxon (rows) in each pairwise year comparison (columns). Taxa are ordered from highest (top) to lowest (bottom) mean contribution across all comparisons. Fill color indicates the taxon's average contribution to dissimilarity for each year-pair comparison (square-root scaled).
